# LLMs as Representation Generators for Gradient‐Free Scientific Optimization: A Case Study in Battery Fast Charging

**DOI:** 10.1002/advs.78012

**Published:** 2026-10-11

**Authors:** Ge Lei, Ferran Brosa Planella, Sterling G. Baird, Samuel J. Cooper

**Affiliations:** ^1^ Dyson School of Design Engineering Imperial College London London UK; ^2^ The Faraday Institution Harwell Campus Didcot UK; ^3^ Mathematics Institute University of Warwick Gibbet Hill Road Coventry CV4 7AL UK; ^4^ Acceleration Consortium University of Toronto Toronto Ontario Canada

**Keywords:** computer science, language model, optimization problem

## Abstract

Scientific optimization problems are often characterized by slow, expensive, and non‐differentiable evaluations, which severely limit efficient exploration of rich design spaces. In these settings, performance depends not only on the optimizer itself, but also on whether the chosen search space can represent good solutions and whether important design requirements are effectively incorporated into the search. Here, we introduce a gradient‐free optimization framework based on large language models (LLMs) that enables the generation and refinement of executable control policies directly from natural language. We propose two complementary paradigms: Prompt‐to‐Optimizer (P2O), which combines LLM‐based structural generation with gradient‐free parameter optimization, and Prompt‐to‐Protocol (P2P), which directly synthesizes fully specified control policies. We apply this framework to battery fast‐charging protocol design, a representative slow‐feedback optimization problem. Across multiple case studies, our methods outperform conventional approaches restricted to predefined functional forms. In a realistic fast‐charging scenario, both approaches improve battery state of health by approximately 4.2% over a representative baseline, with P2P achieving this under matched evaluation budgets. More broadly, our results suggest that LLMs can serve as representation generators that expand the hypothesis space of scientific optimization, enabling efficient search in domains where evaluations are costly, and gradients are unavailable.

## Graphical abstract

Large language models serve as representation generators for gradient‐free scientific optimization. Two complementary approaches produce executable battery‐charging policies either directly or through LLM‐designed neural representations coupled to Bayesian optimization. Under limited evaluation budgets, evaluator feedback guides policy refinement and identifies adaptive fast‐charging protocols that outperform predefined functional forms in physics‐based battery simulations, reducing the need for manual representation design.

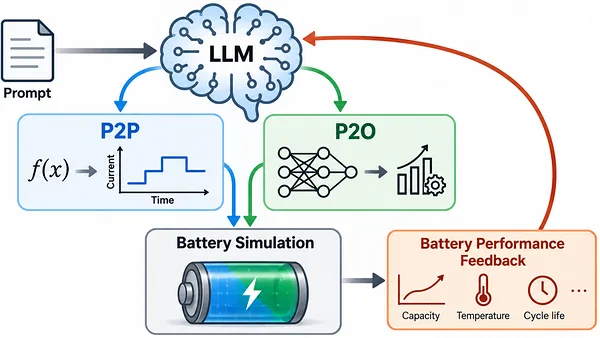

## 1 Introduction

Scientific optimization problems are often characterized by slow, expensive, and non‐differentiable evaluations, which makes it difficult to efficiently explore rich design spaces. In such settings, the challenge lies not only in optimizing candidate solutions, but also in designing suitable solution representations, structural priors, and constraints, and in incorporating high‐level requirements into the optimization process. As a result, conventional approaches typically rely on predefined parameterizations or rigid templates, which impose strong inductive biases and often limit the diversity of solutions that can be discovered. Large language models (LLMs) offer a different route. Rather than operating only within a fixed, human‐designed search space, they can generate executable algorithms and controllers directly from high‐level language specifications, incorporate qualitative feedback, and iteratively refine candidate solutions using natural language descriptions of failures and constraints. This can reduce the need for artisanal function specification, which is often a major bottleneck in the optimization of complex scientific and engineering systems.

In this work, we instantiate this idea in the context of battery charging protocol design. The optimization of battery charging protocols is fundamental to next‐generation electric mobility, because the charging protocol governs how energy is delivered into the cell and directly influences both charging efficiency and long‐term battery durability [1, 2]. Poorly designed protocols can accelerate degradation or create thermal and mechanical stresses that reduce battery lifespan. This challenge becomes even more pronounced during fast charging: although it shortens downtime, it significantly intensifies degradation mechanisms such as lithium plating [3], side reactions, and thermal runaway [4, 5]. These efficiency‐aging trade‐offs highlight the need for charging protocols that achieve rapid charging while maintaining battery longevity, a requirement for reliable and high‐performance electric mobility.

However, optimizing battery charging protocols remains a long‐standing challenge due to the vast design space and the nonlinear, history‐dependent nature of battery dynamics. The difficulty is compounded by the slow and costly nature of physical experiments and high‐fidelity simulations, which limits how quickly protocols can be tested and improved. Consequently, many existing studies impose strong structural constraints on the protocol class, reducing the search space to make the optimization tractable. Analytical forms such as exponential or sigmoid curves [6, 7] are easy to optimize but impose strong inductive biases and limit strategy diversity. Piecewise‐constant controls [8, 9] offer more flexibility, but too few segments oversimplify the space, whereas too many can create jagged profiles and high‐dimensional non‐convex problems under limited evaluation budgets. More detailed physics‐based approaches have also computed fast‐charging protocols directly from high‐fidelity porous‐electrode models using dynamic optimization or hybrid simulation frameworks (e.g., the MPET‐based protocol design of Galuppini et al. [10]), but these methods still require the researcher to specify an operating‐mode structure in advance.

LLMs are widely recognized for their language skills [11], and recent work has shown that they can also support algorithm and program search, most notably in “FunSearch” [12, 13, 14, 15]. Compared to conventional methods, LLMs offer several notable advantages: (1) they can interpret rich, language‐based feedback instead of relying solely on scalar rewards; (2) their strong generative ability allows exploration beyond predefined search spaces; and (3) their broad pretraining may provide useful priors for proposing initial candidate structures.

LLM‐enabled battery research is also developing rapidly. Zhao et al. surveyed the potential of LLMs to synthesize dispersed battery knowledge and demonstrated literature‐assisted analysis of fast charging [16]. Leng et al. subsequently introduced ChatSSB, a retrieval‐augmented, multi‐agent scientific assistant for solid‐state‐battery literature analysis and quantitative research support [17]. Zhong et al. developed BatteryAgent, which coordinates specialized agents and computational tools for interdisciplinary analysis across materials and cell‐level design [18]. These studies primarily position LLMs as knowledge interfaces, scientific assistants, or reasoning agents. In contrast, the present work uses an LLM to generate executable charging‐control representations and protocols inside a closed‐loop optimization process, with every candidate judged by a physics‐based battery evaluator.

Although several recent studies have demonstrated the ability of LLMs to iteratively improve algorithms, these demonstrations largely rely on settings where algorithm evaluations are extremely fast, enabling the model to perform thousands of trial‐and‐error iterations. In applications such as battery charging protocol optimization, however, each evaluation is slow, expensive, and often experimentally constrained. It has therefore not previously been established that LLM‐based optimization can deliver meaningful improvements under a limited feedback budget.

A second concern is numerical precision. As language‐based models, LLMs excel at structural abstraction, but they can be unreliable for arithmetic and quantitative precision [19, 20, 21]. We therefore do not assume that the LLM performs reliable numerical training. Instead, we study two ways of assigning roles between the LLM and the numerical evaluator. In the first approach, the LLM directly proposes a complete executable charging policy, including numerical parameters; these values are treated only as candidate components, and the battery evaluator determines whether the resulting policy performs well. In the second approach, the LLM proposes only the policy representation, while a dedicated black‐box optimizer tunes its numerical parameters. These two approaches correspond to Prompt‐to‐Protocol (P2P) and Prompt‐to‐Optimizer (P2O), respectively, which are introduced in detail below.

To investigate these two roles, we introduce two LLM‐assisted paradigms: Prompt‐to‐Protocol (P2P; single loop) and Prompt‐to‐Optimizer (P2O; two loops), as shown in Figure 1. P2P asks the LLM to generate complete executable charging protocols in a single loop. Each generated protocol is evaluated by the battery model, and better‐performing candidates are retained through population‐based selection. P2O separates structural proposal from numerical optimization: the LLM iteratively designs and refines compact neural‐network‐based policy representations, while the numerical parameters of each proposed representation are optimized using a gradient‐free Sparse Axis‐Aligned Subspace Bayesian Optimization (SAASBO) inner loop [22, 23]. Together, these methods allow us to test whether LLMs are more useful as direct protocol generators or as representation generators coupled to a dedicated black‐box optimizer.

**FIGURE 1:**
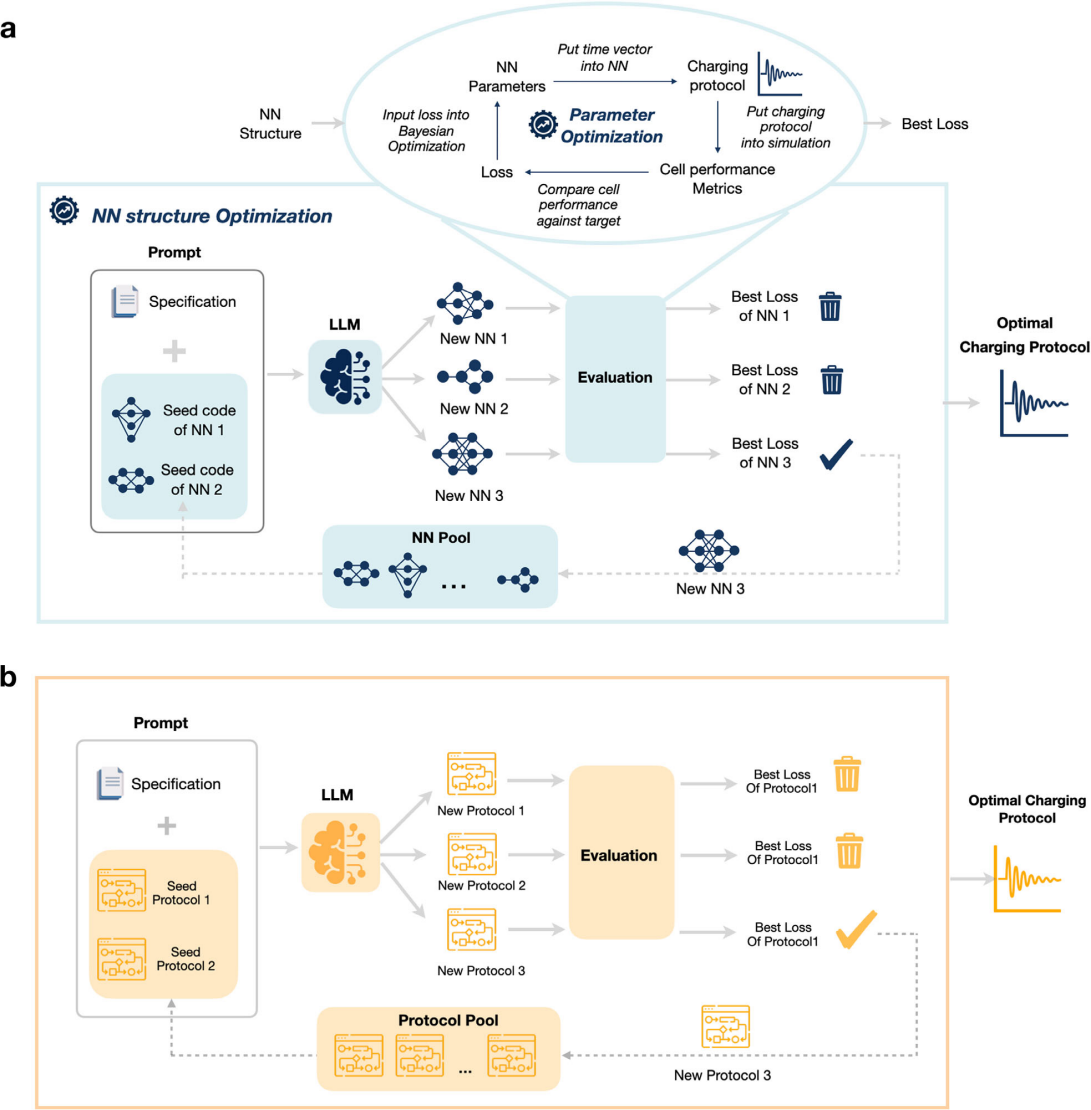
Overview of the proposed LLM‐assisted optimization frameworks. (a) P2O (two loops): LLM‐guided neural network generation and refinement with a SAASBO‐based inner optimization loop. (b) P2P (single loop): direct LLM generation of explicit charging protocols without inner‐loop optimization.

To evaluate the proposed methods across progressively more battery‐relevant simulation settings, we conduct three case studies: predefined heat search, constant‐heat protocol search, and degradation‐aware fast‐charging protocol search. These case studies are designed to test different aspects of the framework: whether LLMs can propose useful policy representations, whether numerical tuning should be delegated to a black‐box optimizer, and whether the resulting policies can improve a simulated battery fast‐charging objective under a limited evaluation budget. In the outer‐loop architecture evolution tasks, LLM‐guided P2O outperforms Bayesian optimization, evolutionary algorithms, and random search under the chosen search space and evaluation budget. In the degradation‐aware fast‐charging simulation, P2P performs strongly within the matched evaluation budget, showing that direct LLM‐generated numerical candidates can be effective when combined with evaluator‐based selection. However, this should be interpreted as successful black‐box candidate generation rather than precise numerical training by the LLM. P2O provides a more systematic route when additional evaluations are available: by delegating numerical parameter tuning to SAASBO, it can further refine LLM‐generated policy representations and reach the best overall performance after additional inner‐loop optimization. Overall, these results suggest that LLM‐assisted representation search can provide a complementary route to conventional parameterized protocol optimization in simulation, particularly when the feedback budget is limited.

## 2 Methods

A key design question is how to leverage LLMs when the evaluator is expensive, non‐differentiable, or treated as a black box. We use the term “evaluator” to refer either to a simulator, as in this paper, or to a physical cell‐testing workflow in future experimental use. The LLM never receives gradients from this evaluator. It proposes executable policy code, and the evaluator returns a scalar loss after applying the policy under the specified constraints.

Motivated by the limited numerical reliability of LLMs, we study two complementary paradigms for representation‐driven optimization. The first, Prompt‐to‐Optimizer (P2O; two loops), uses an LLM to generate and iteratively refine executable solution structures, while a gradient‐free optimizer tunes their parameters in an inner loop. The second, Prompt‐to‐Protocol (P2P; single loop), allows the LLM to directly generate fully specified executable policies, including both structural form and numerical parameters. Together, these two paradigms span a spectrum between hybrid structure‐and‐parameter optimization and fully language‐guided policy proposal.

### 2.1 Prompt‐to‐Optimizer (P2O)

Neural networks are expressive representations for smooth and state‐adaptive control policies. Their outputs vary continuously with respect to their parameters, so small parameter changes can produce gradual changes in the generated current profile. However, ordinary backpropagation requires a differentiable and stable objective. Physical charge–discharge experiments do not provide such gradients, and many battery‐simulation workflows include solver events, voltage cut‐offs, termination conditions, and safety filters that are most naturally treated as black‐box evaluations. We therefore adopt a gradient‐free formulation for compatibility with simulator‐in‐the‐loop and future experiment‐in‐the‐loop settings.

ALGORITHM 1 **P2O (two‐loop) evolutionary optimization.**

**Table:** 

**Require**: Initial pool $P_{0}$ (set of (s,θ,L) tuples or architectures with initialized losses); generations T; children per generation C; pool size N; SAASBO budget B	
**Ensure**: Best network structure and parameters	
1:	$P\leftarrow P_{0}$
2:	**for** t=1 to T **do**
3:	C←∅ ▹ reset child set
4:	**for** c=1 to C **do**
5:	Select parents $p_{1},p_{2}$ from P ▹ e.g. tournament selection
6:	$s_{c}\leftarrow \mathrm{LLMGENERATE}\left(p_{1},p_{2}\right)$
7:	$\left(\theta_{c}^{*},L_{c}\right)\leftarrow \mathrm{SAASBOOPTIMIZE}\left(s_{c},B\right)$ ▹ optimize parameters via SAASBO for B evaluations
8:	$C\leftarrow C\cup \{\left(s_{c},\theta_{c}^{*},L_{c}\right)\}$
9:	P←SELECTTOPN(P∪C,N) ▹ keep N best networks by loss
10:	**return** $\left(s^{*},\theta^{*}\right)$ ▹ where $\left(s^{*},\theta^{*},L^{*}\right)=\arg\min_{(s,\theta ,L)\in P}L$

Even when gradients can in principle be obtained from simulations, they may be inconvenient or unreliable for the present workflow. Complex physical models can involve stiff ODE/PDE dynamics [24], long‐horizon differentiation through implicit solvers can demand substantial memory and computation [25], and safety constraints or termination conditions can induce abrupt mode transitions [26, 27]. These considerations motivate a black‐box optimizer for the inner loop.

We choose SAASBO because it provides sample‐efficient optimization of continuous parameters without requiring gradients, making it suitable for the small neural networks used here and compatible with both black‐box simulation and future experiment‐in‐the‐loop settings.

This choice should not be read as a claim that gradient‐based control, reinforcement learning, surrogate modeling, or model‐predictive control cannot be applied to battery charging. Those approaches are appropriate when a differentiable simulator, a trusted reward model, or a sufficiently large interaction budget is available. Our objective is complementary: to test whether an LLM can propose compact policy representations that can be optimized and compared using only black‐box evaluations. For small neural networks (e.g., fewer than 35 parameters), gradient‐free optimization methods such as SAASBO provide a practical parameter‐tuning mechanism. Although each small network may have limited expressiveness, evolving a diverse set of small architectures over time enables exploration of a broader solution space while preserving optimization stability.

Specifically, we propose an evolutionary optimization framework consisting of two nested loops: an outer loop that employs LLM‐based neural architecture generation, and an inner loop in which the network parameters are optimized with SAASBO, as illustrated in Figure 1. The process begins with an LLM generating an initial pool of neural network architectures based on a prompt that specifies several design considerations, including the constraint that each network should contain fewer than 35 trainable parameters. These architectures correspond to candidate solution structures expressed as executable Python code. At each subsequent generation, two high‐performing networks are sampled from the current pool (e.g., via tournament selection) to serve as parents. Conditioned on these parent architectures, the LLM proposes new child architectures by recombining and modifying their structural features.

In the inner loop, for each newly generated architecture, its trainable parameters are optimized using SAASBO, a Bayesian optimization method designed to remain effective in high‐dimensional gradient‐free settings. After optimization, the resulting performance metric (best loss) is compared against those of the architectures currently in the pool. If a new network outperforms existing members, it is retained, while weaker candidates are progressively removed. Through this iterative process of generation, optimization, and selection, the performance of the architecture pool gradually improves. The complete procedure is summarized in Algorithm 1. The detailed prompt is provided in Appendix B.2.

### 2.2 Prompt‐to‐Protocol (P2P)

Unlike P2O (two loops), P2P is a single‐loop scheme: the LLM directly proposes an executable charging protocol with an (arbitrary) explicit function, including its parameter values, based on a prompt containing various relevant pieces of information about desirable behavior.

ALGORITHM 2 **P2P (single‐loop) direct algorithm generation.**

**Table:** 

**Require**: Initial pool $P_{0}$ (set of (s,L) pairs with pre‐evaluated losses); number of generations T; children per generation C; pool size N	
**Ensure**: Best algorithm/protocol $s^{*}$	
1:	$P\leftarrow P_{0}$
2:	**for** t=1 **to** T **do**
3:	C←∅ ▹ initialize child set
4:	**for** c=1 **to** C **do**
5:	Select parents $p_{1},p_{2}$ from P ▹ e.g. tournament selection
6:	$s_{c}\leftarrow \mathrm{LLMGENERATE}\left(p_{1},p_{2}\right)$ ▹ LLM proposes a new protocol
7:	$L_{c}\leftarrow \mathrm{EVALUATE}\left(s_{c}\right)$ ▹ simulation/experiment returns loss
8:	$C\leftarrow C\cup \{\left(s_{c},L_{c}\right)\}$
9:	P←SELECTTOPN(P∪C,N) ▹ retain N best candidates by loss
10:	**return** $s^{*}$ ▹ $\left(s^{*},L^{*}\right)=\arg\min_{(s,L)\in P}L$

Initially, a pool of candidate LLM‐generated protocols is created and their loss is evaluated by the chosen evaluator. In this paper the evaluator is a battery simulation; in an experimental implementation it would be a cell‐cycling test under the same safety constraints. At each subsequent generation, a subset of parent algorithms is selected from the current pool using tournament selection. The LLM is then prompted to generate new child candidates based on these selected parents and their observed performance. Each child algorithm is evaluated to obtain a scalar loss. Invalid code, protocols that violate hard constraints, or protocols that fail to reach the target SOC within the allowed time are rejected or penalized by the evaluator. If a child outperforms an existing member of the pool, it replaces a weaker one. This cycle of selection, generation, evaluation, and replacement continues until the trial budget is exhausted or the population converges.

Because P2P relies on candidate generation rather than continuous numerical descent, its strength is rapid exploration of qualitatively different protocol forms when the prompt contains useful semantic constraints, such as voltage limits and time targets. It is less appropriate when success depends mainly on precise tuning of many continuous parameters; in such cases P2O's separation between structural proposal and numerical optimization is preferable. The complete procedure is summarized in Algorithm 2.

### 2.3 Non‐LLM Benchmarks

In the first case study, the performance of the P2O approach is compared against three conventional gradient‐free strategies for specifying neural‐network architectures: Genetic Algorithms, Bayesian Optimization, and Random Search. In each non‐LLM benchmark, the architecture search space must be predefined by the user (e.g., variables controlling the number of layers, activation functions, and optional normalization layers). This comparison is designed to isolate the value of LLM‐based representation proposal under a common black‐box evaluation budget. It is not intended to exhaust all possible learning‐based control methods, such as policy‐gradient reinforcement learning or differentiable surrogate‐control approaches, which require different assumptions about simulator access, reward modeling, and interaction budget.

### 2.4 Battery Modeling

We model battery behavior using a physics‐based Doyle–Fuller–Newman (DFN) electrochemical model augmented with degradation and thermal dynamics, implemented through PyBaMM [28, 29]. The DFN model resolves lithium diffusion in solid particles, electrolyte transport, charge‐transfer kinetics, and voltage response under arbitrary current profiles, allowing protocol‐dependent ageing phenomena such as SEI growth, loss of active material, and particle cracking to be represented O'Kane et al. [30]. To reflect cycling conditions, we couple the electrochemical model to a lumped thermal model that accounts for ohmic, kinetic, and entropic heat generation as well as heat dissipation to the environment. Degradation is simulated using reaction‐limited SEI growth with porosity change, stress‐driven electrode damage, and cracking submodels. Because full lifetime simulation is computationally expensive, we accelerate ageing by increasing selected degradation‐rate parameters while checking that protocol rankings remain consistent under non‐accelerated conditions.

## 3 Results

We evaluate the proposed framework through a series of simulation case studies in battery charging protocol design. These case studies are designed to progressively test different components of the framework before applying it to a degradation‐aware fast‐charging objective. Case 1, the predefined‐protocol search, is a controlled and simplified setting used to test the effectiveness of the outer‐loop representation search in P2O and has a differentiable loss. Case 2, the constant‐heating protocol search, introduces a black‐box non‐differentiable setting and focuses on validating the inner‐loop SAASBO optimizer while also comparing with P2P. Case 3 is the most application‐oriented setting: discovering an adaptive fast‐charging protocol in a DFN model with thermal and degradation submodels.

All case studies use PyBaMM [28] as the evaluator, allowing the proposed optimization methods to be compared under a fixed battery model, fixed constraints, and matched evaluation budgets. Our aim is to validate the search framework rather than to certify the discovered protocols for direct experimental deployment. The simulations test whether LLM‐assisted search can generate executable charging policies, use evaluator feedback to improve them, and operate effectively under limited evaluations. Applying the framework to physical cells would require model calibration, safety screening, and cell‐level validation, as discussed below.

All experiments are conducted using GPT‐5‐2025‐08‐07 as the LLM, using the default inference configuration, where the reasoning effort is set to medium. We intentionally keep prompts constraint‐focused (operational limits such as voltage/current bounds and time targets) and do not provide cell‐specific identity information (e.g., chemistry, a particular parameter set, or fitted cell models). This choice is intended to reduce reliance on cell‐specific priors or handcrafted heuristics encoded in the prompt, and to better reflect deployment settings where the evaluator (simulation or experiment) provides the task‐specific feedback.

No LLM‐generated protocol or network architecture was manually corrected. Nearly all candidates (>99%) were executable under the provided templates; invalid candidates were automatically penalized in P2P or discarded in P2O rather than repaired.

### 3.1 Case 1: Predefined Heat Search

We begin with a deliberately simplified setting to isolate and evaluate the effectiveness of the outer‐loop neural architecture optimization in the P2O (two‐loop) framework. The predefined targets in this case are not meant to represent realistic charging protocols; rather, they create a controlled environment where the influence of the outer loop can be examined without confounding factors. To eliminate interference from the inner loop, we adopt a stable and fully differentiable setup in which the inner‐loop neural network parameters are optimized using standard gradient descent.

Under this simplified setting, the task is to recover two predefined profiles ‐ a complex explicit function and a simple constant‐heating pattern. This allows us to assess how effectively the outer loop can guide architecture evolution. To contextualize the performance of P2O, we compare it against common baselines such as Bayesian Optimization (BO), Genetic Algorithm (GA), and Random Search. We also include a preliminary comparison with P2P (single loop), which, unlike P2O, is entirely gradient‐free since it directly generates explicit protocols without an inner‐loop optimizer.

Since Case 1 aims only to examine outer‐loop optimization rather than full physical fidelity, we use a simple Equivalent Circuit Model (ECM) combined with a lumped thermal model (see Appendix C.1) to evaluate protocol performance and enable gradient‐based training of the neural network applied to a non‐linear system. Model performance is measured using the mean squared error (MSE) between the predicted and target heat profiles:

(1) $$L=\frac{1}{N}\sum_{i=1}^{N}{\left(Q_{\text{pred},i}-Q_{\text{target},i}\right)}^{2}$$

In the LLM‐guided variant, LLM initially generates ten diverse seed architectures and then three offspring architectures per seed at each iteration to promote exploration (prompt templates in Appendix B.2). The LLM is encouraged to explore broadly rather than follow fixed layer‐width patterns. To maintain compactness, it is explicitly instructed to propose lightweight models, and any design exceeding 35 trainable parameters is discarded before evaluation. The BO, GA, and Random Sampling variants use Optuna‐guided sampling [31], a standard genetic algorithm, and independent random proposals, respectively. Their search space is restricted to compact multilayer perceptrons (MLPs) with at most 3–4 hidden layers, 1–5 neurons per layer, varied activation functions, and optional normalization layers.

For all four variants, each candidate network maps 1000 uniformly sampled time steps (1–3600 s) to a 1000‐step current profile, which is then evaluated using a battery simulation model to compute heat generation. The 35‐parameter upper bound is applied uniformly across all variants (see Appendix C.2).

P2P (single‐loop) operates without an inner optimization loop. Instead of training, it directly prompts the LLM to generate an explicit time‐dependent current function, I(t), that serves as the charging protocol. The prompt (see Appendix B.1) specifies only the desired protocol behavior and the constant‐heat requirement, without exposing any battery‐model details. The LLM produces ten diverse seed functions, along with three offspring variants in each iteration. All generated functions are evaluated directly, with no further optimization.

In both predefined explicit function and constant heating rate experiments, the LLM‐guided P2O (two‐loop) framework performs strongly in the outer loop. Figure 2a–c presents the results for the complex predefined‐function task, while Figure 2d–f presents the constant‐heating results. For the complex predefined function task, the LLM‐guided search reduces loss by nearly two orders of magnitude within 20 iterations, outperforming baselines after five evaluations. This early advantage likely reflects its ability to propose architectures informed by prior knowledge. BO shows comparable early progress but higher variance, EA improves slowly with a single large gain near iteration 45, and Random search remains one order of magnitude worse. In contrast, performance differences are less pronounced in the simpler constant‐heating case. Because the target profile is structurally simple and imposes minimal demands on network architecture, EA, BO, and the LLM‐guided approach all perform competitively. BO shows stable convergence but can become trapped in local minima, whereas both EA and the LLM reach similarly low loss values.

**FIGURE 2:**
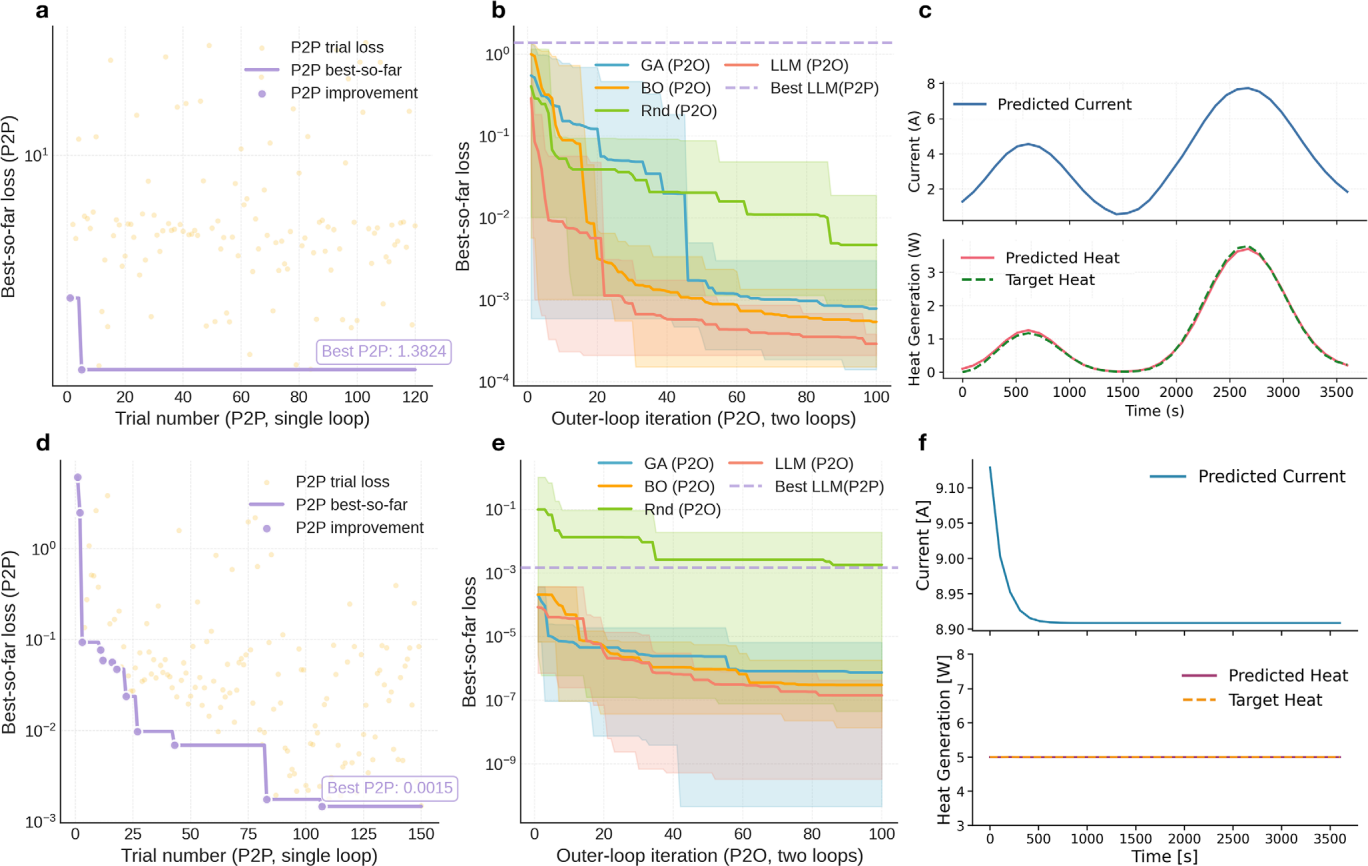
Optimization results for P2O (two loops) and P2P (single loop) across two tasks. (a–c) Complex predefined protocol; (d–f) constant‐heating protocol. (a, d) show P2P performance, where LLM directly generates explicit charging functions. (b, e) compare P2O variants using different outer‐loop mechanisms—LLM‐guided, BO, GA, and Random Sampling; each curve shows the cumulative best loss over iterations, where the solid line denotes the mean across 10 runs, and the shaded area marks the range between the minimum and maximum best losses; the LLM‐guided approach converges fastest and achieves the lowest loss (dashed lines mark best P2P results). (c, f) display predicted current and heat generation profiles of the best‐performing protocols.

In complex predefined function tasks, P2P (single‐loop) performs notably worse than P2O (two‐loop). Under simple constant‐current protocols, P2P achieves a best loss of about $1.5\times 10^{-3}$, which is comparable to a gradient‐trained neural network in this controlled setting. This result is consistent with the intended role of P2P: it can be effective when the desired behavior is simple and can be described clearly in language, but it is less reliable for irregular numerical profiles when the prompt provides little structural guidance. The P2O variants in Case 1 use gradient‐based inner‐loop optimization and therefore serve primarily to isolate the effect of outer‐loop representation search rather than to provide a matched‐budget comparison with P2P. Detailed comparisons are presented in the next section.

The LLM‐guided variant also probes a less rigid architecture space than the non‐LLM benchmarks. Genetic algorithms, Bayesian optimization, and random search can of course be applied to richer architecture spaces if those spaces are specified in advance, but that specification must be supplied by the researcher. In our benchmarks, the non‐LLM methods search over a compact, predefined set of layer counts, widths, activation functions, and normalization choices. The LLM, by contrast, can propose executable motifs outside this manually enumerated space, such as alternative residual or gated structures, while still being filtered by the same parameter‐count and evaluation rules. The advantage observed here should therefore be interpreted as a reduction in representation‐engineering burden, not as a claim that conventional optimizers are intrinsically unable to search richer spaces.

The optimal neural architectures obtained in the constant‐heating setting differ in structure, ranging from relatively complex multi‐branch networks to simpler feed‐forward layers and compact residual units with smooth nonlinearities. The first two architectures correspond to larger search spaces. In contrast, the last one induces a much smaller family of protocol shapes; however, its typical forms closely match the optimal protocol's decreasing–then–flattening pattern (Figure 2f), which in turn enables strong optimization performance. Detailed visualizations of the associated search spaces are provided in Appendix C.4 (Figure C1).

**FIGURE 3:**
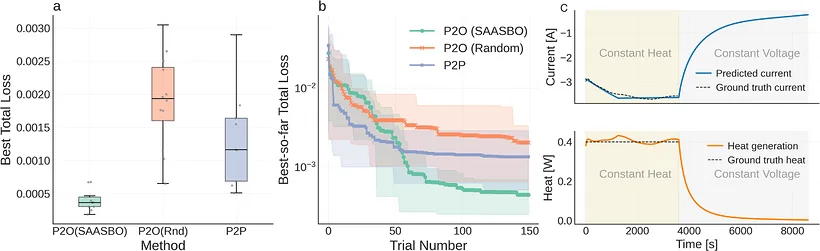
Optimization under a gradient‐free setting. (a) Best final loss across ten runs of SAASBO and Random Search (inner‐loop optimizers of P2O) and the LLM‐based P2P method (single‐loop). (b) Convergence of the best‐so‐far loss over battery‐simulation trials for all three approaches. (c) Verification of the optimized charging protocol obtained from SAASBO.

**FIGURE 4:**
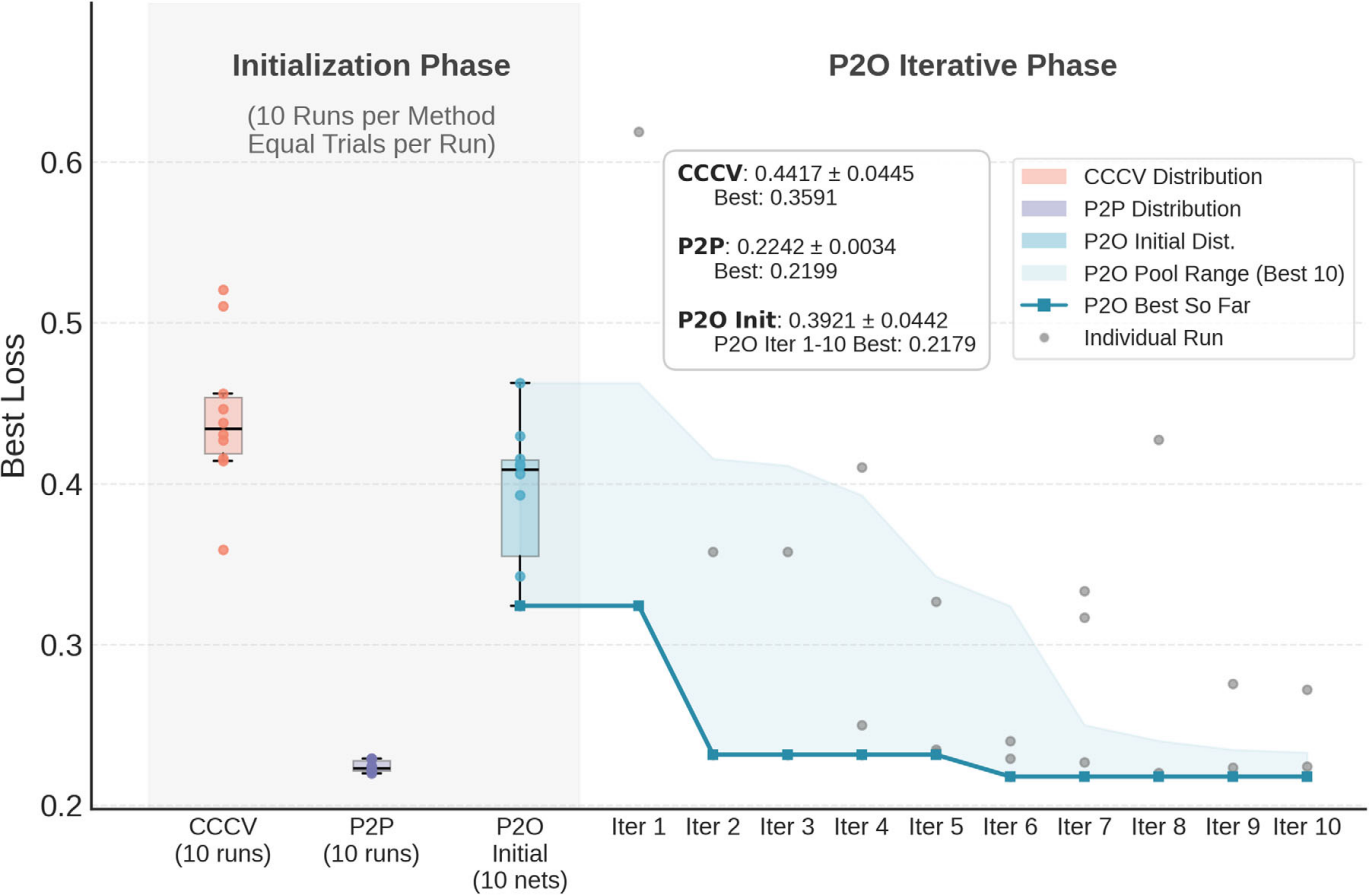
Best‐loss comparison under controlled evaluation budgets. We define the budget as the number of full protocol evaluations used to compute the Case 3 loss. Left: matched‐budget comparison between CCCV and P2P (10 runs each, 210 evaluations per run) and the P2O initialization phase (10 evaluated neural architectures), showing variability across independent runs/candidates under the same evaluation count. Right: illustrative trajectory of P2O when additional evaluations are permitted beyond the matched‐budget regime, showing that outer‐loop evolution can further improve performance with extra evaluation budget. (Only the left panel represents a matched‐budget comparison).

**FIGURE 5:**
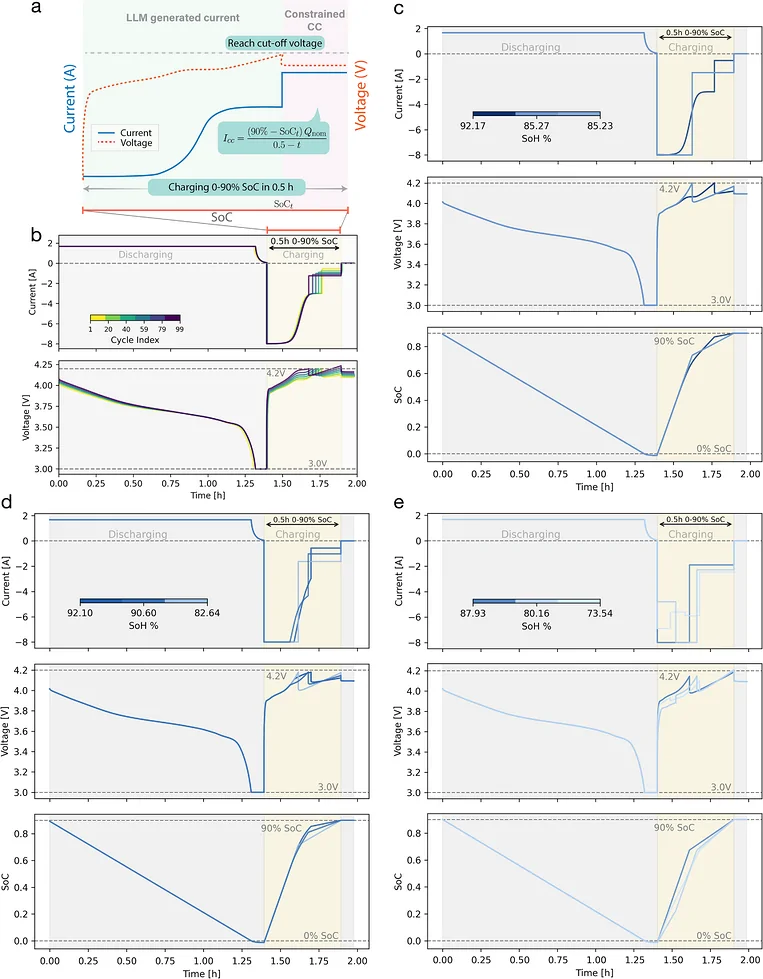
Comparison of LLM‐generated charging protocols with conventional baselines. (a) Charging‐phase schematic: the LLM generates a variable‐current profile until the cut‐off voltage, followed (if needed) by a constrained CC phase to reach the target SOC within the time limit. (b–e) Full charge–discharge trajectories; the yellow region marks the charging phase (LLM segment + constrained CC). (b) Best P2O protocol across cycles, showing earlier voltage‐limit entry with degradation. (c) P2O, (d) P2P, and (e) CCCV: best result with representative best, worst, and intermediate trajectories.

**FIGURE C1:**
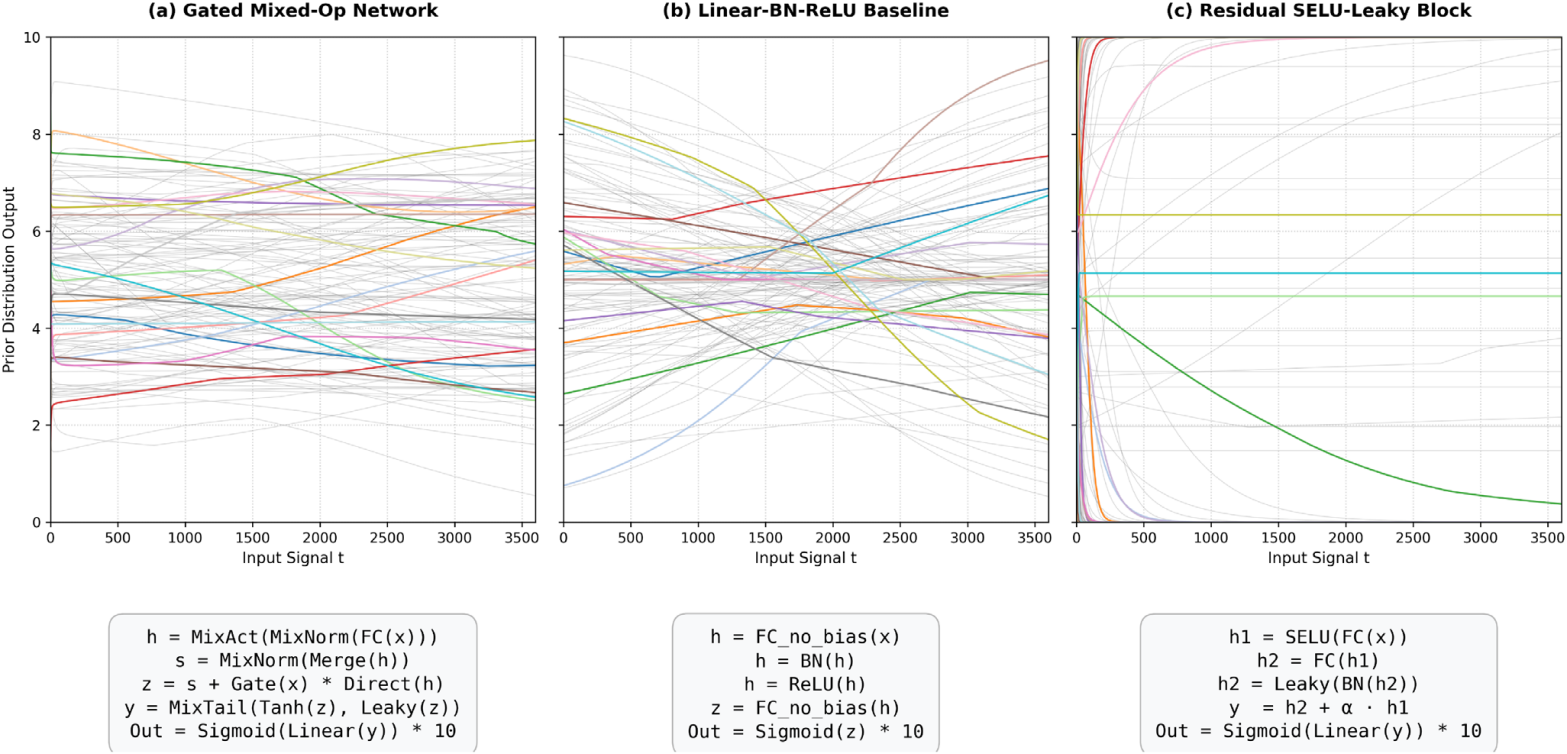
Visualization of architectural functional priors (search spaces). The plots illustrate the outputs of randomly initialized networks (N=120) over the input signal t. (a) The Gated Mixed‐Op Network exhibits a dense and highly expressive search space. (b) The Linear‐BN‐ReLU Baseline shows a large search space with characteristic piecewise‐linear behavior. (c) The Residual SELU‐Leaky Block demonstrates a more restricted functional prior, visually consistent with the shape of the constant‐heating protocol in this battery model.

### 3.2 Case 2: Constant Heat Protocol Search

In practice, battery research relies on experimental validation, which is inherently non‐differentiable. This case study therefore considers a non‐differentiable setting to evaluate the inner‐loop optimization of P2O (two loops) and compare it with P2P (single loop). The study aims to identify a charging protocol that maintains a constant heat generation rate of 0.4 W over 1 h charge, employing a DFN model coupled with a lumped thermal model. The MSE between the predicted and target heat serves as the evaluation metric.

A compact neural network identified from Case Study 1 is used for testing the inner loop. The neural network's parameters are optimized using SAASBO and Random search, each repeated ten times. The P2P (single loop) method is also tested, where the LLM directly evolves explicit charging protocols. All approaches are compared under equivalent battery simulation costs or trial counts, which dominate computational expense in practical scenarios.

As shown in Figure 3a,b, P2O using SAASBO consistently outperforms random search, demonstrating superior convergence stability. The P2P results fall between those of SAASBO and random search, which is expected because SAASBO is a dedicated parameter optimization algorithm, whereas the LLM in P2P relies on heuristic reasoning based on linguistic cues. Although the prompt did not include explicit physical guidance on how to maintain a heat generation rate of 0.4 W, P2P still achieved effective optimization within 150 trials and improved particularly rapidly during approximately the first 50 evaluations. P2P can change both the functional form and numerical parameters of a complete protocol from one trial to the next, enabling large moves through the search space before a detailed surrogate has been learned. By contrast, P2O keeps the protocol representation fixed and uses its early observations to fit and update the SAASBO surrogate over continuous parameters. P2P therefore has an early exploration advantage, whereas SAASBO becomes more effective as observations accumulate and ultimately provides more systematic numerical refinement and a lower final loss.

The best‐performing protocol generated by the LLM in the P2P approach is shown in Appendix D.2. When asked to explain its proposed method, the LLM indicated that the approach was not derived from any particular existing literature or a clear physical principle. Upon closer inspection, this protocol appears to be a problem‐specific heuristic refined through iterative changes to both parameters and structural components. Its resulting current trajectory is nevertheless physically reasonable: the increasing current magnitude during the early stage can compensate for the evolving electrochemical resistance, polarization, and thermal state as SOC and temperature change, while the later leveling reflects the more gradual variation in the current required to maintain 0.4 W of heat generation. The independently computed reference current exhibits the same increasing‐then‐leveling trend (Figure 3c), supporting this trajectory‐level interpretation. The individual nonlinear terms of the generated expression cannot, however, be assigned a one‐to‐one mechanistic meaning. The exact protocol is a problem‐specific heuristic and may not transfer directly to different cells or operating conditions; for related systems it is better regarded as a simulation‐informed initialization for subsequent model recalibration and experimental refinement. Overall, these results reinforce the interpretation of P2P as heuristic black‐box candidate generation. When the prompt lacks information that can be directly used for algorithm design, P2O (two loops) provides more reliable optimization through dedicated parameter tuning, although at the cost of additional evaluations.

To validate whether our optimization algorithm has learned a near‐optimal charging protocol, we compute the reference current (based on PyBaMM's differential stepping) that exactly maintains 0.4 W of heat generation and compare it with the optimized result (N.B. this reference protocol is not used during optimization). As shown in Figure 3c, the optimized current closely matches the constant‐heat reference, demonstrating that the learned protocol captures the desired thermal behavior. Naturally, this ground truth analysis cannot be performed on real experiments or under more complex simulation conditions.

### 3.3 Case 3: Adaptive Fast Charging Protocol Search

This case study is the closest in the paper to a practical fast‐charging setting, while remaining entirely simulation‐based. The objective is to identify a fast‐charging strategy that minimizes simulated battery degradation over the same number of charge cycles. We use “fast charging” in a cell‐level sense: the protocol must charge a 2.5 Ah cell from 0 to 90% SOC in 0.5 h, corresponding to an average rate of approximately 1.8 C. To ensure a fair comparison among different charging protocols, we follow the principle used by Attia et al. [8] by fixing the total charging time required to reach a target SOC. In their work, this was achieved using a protocol composed of three constant‐current (CC) segments followed by a constrained CC phase. In contrast, our method uses either an LLM‐generated neural network to produce a flexible state‐dependent current profile or an LLM‐generated direct protocol, both of which enable broader exploration of the policy space. Once the cell voltage approaches the cut‐off threshold, a constrained CC phase is applied to ensure that the target SOC is achieved within the allotted charging time (see Figure 5a).

In real charging scenarios, a battery may begin at different voltages, temperatures, and states of charge, rather than from a fixed initial condition. Therefore, we adopt an adaptive charging scheme in which the neural network or explicit function maps the instantaneous voltage, temperature, and SOC to the output current. This setup differs from Case Study 1, where the charging protocol is a direct time‐to‐current mapping. At each time step, the network or algorithm receives the current battery state and outputs the charging current, which is then applied to the battery model; the updated state at the next step is used as input for subsequent predictions. The neural network architecture is flexible and may take various forms, including multilayer perceptrons (MLPs), recurrent neural networks (RNNs), or other lightweight structures generated by the LLM.

In this case study, each simulation cycle consists of five stages: (i) discharge at a constant current of C/3 until the cell voltage reaches 2.5V; (ii) voltage hold at 2.5V until the current decreases below 50mA; (iii) neural‐network‐controlled charging, where the current is given directly by the neural network as a function of the instantaneous voltage, temperature, and SOC, and applied until one of the following conditions is met: V=4.20V, SOC=90%, or $t=t_{\max}=0.5\;h$; (iv) constrained CC charging (if required), where, if SOC<90% and time remains, the constant current is set to

(2) $$I_{\mathrm{cc}}=-\frac{\left({\mathrm{SOC}}_{\mathrm{tgt}}-\mathrm{SOC}\right)\;Q_{\mathrm{nom}}}{t_{\mathrm{rem}}}$$

and applied until either the target SOC is reached; otherwise, if the time limit is reached first, the protocol is discarded. Here $I_{\mathrm{cc}}$ is the imposed constant charging current, ${\mathrm{SOC}}_{\mathrm{tgt}}=90\%$ is the target state of charge, SOC is the instantaneous state of charge at the start of this stage, $Q_{\mathrm{nom}}$ is the nominal cell capacity, and $t_{\mathrm{rem}}^{h}$ is the remaining charging time; (v) finally, a rest period of 300s at zero current is imposed. The procedure is detailed in Algorithm 3.

ALGORITHM 3 **Battery Cycling with LLM Derived Charging Policy**

**Table:** 

**Require**: nominal capacity $Q_{\text{nom}}=2.5\;\mathrm{Ah}$; target ${\mathrm{SOC}}_{\text{tgt}}=90\%$; maximum charging time $t_{\max}=0.5\;h$; voltage threshold $V_{\text{thr}}=4.20\;V$; rest time 300s; LLM derived charging policy π that maps $\left(V,T_{\text{cell}},\mathrm{SOC}\right)$ to current I	
1:	**for** c=1 to N **do**
2:	**(A) Discharge**: $I\leftarrow Q_{\text{nom}}/3$ until V≤2.5V
3:	**(B) CV tail**: hold V←2.5V until |I|≤0.05A
4:	**(C) Policy controlled charge**: t←0
5:	**while** $t<t_{\max}$ **and** $V<V_{\text{thr}}$ **and** $\mathrm{SOC}<{\mathrm{SOC}}_{\text{tgt}}$ **do**
6:	$I\leftarrow \pi (V,T_{\text{cell}},\mathrm{SOC})$
7:	Apply I for Δt to update V, $T_{\text{cell}}$, and SOC
8:	t←t+Δt
9:	**(D) CC constrained stage (if needed)**:
10:	**if** $\mathrm{SOC}<{\mathrm{SOC}}_{\text{tgt}}$ **and** $t<t_{\max}$ **then**
11:	$t_{\text{rem}}^{h}\leftarrow \left(t_{\max}-t\right)/3600$
12:	$I_{\text{cc}}\leftarrow -\frac{\left({\mathrm{SOC}}_{\text{tgt}}-\mathrm{SOC}\right)Q_{\text{nom}}}{t_{\text{rem}}^{h}}$
13:	**while** $t<t_{\max}$ **and** $\mathrm{SOC}<{\mathrm{SOC}}_{\text{tgt}}$ **do**
14:	Apply $I_{\text{cc}}$ for Δt to update V, $T_{\text{cell}},\mathrm{SOC}$
15:	t←t+Δt
16:	**else if** $\mathrm{SOC}={\mathrm{SOC}}_{\text{tgt}}$ **and** $t\leq t_{\max}$ **then**
17:	skip CC stage
18:	**else**
19:	discard cycle
20:	**(E) Rest**: I←0 for 300s
21:	record SOH

In this case study, we used PyBaMM to simulate the effect of the charging protocols, based on the DFN model plus degradation models. The cell was parameterized using the Ai2020 dataset [32] and equipped with the following degradation submodels: swelling‐induced cracking [33], stress‐driven loss of active material [34], reaction‐limited SEI growth with porosity change [35], and SEI formation on cracks [33]. A lumped thermal model was also included, and all degradation and thermal processes were integrated within the DFN framework as detailed in Appendix E.1.

To accelerate aging and obtain results within a feasible runtime, we increased the degradation rate by modifying several model parameters, most notably by scaling the SEI reaction exchange current density by a factor of five. Additional adjustments were made to electrode loss terms, particle cracking rates, the heat transfer coefficient, and the ambient temperature (see Appendix E.2). In the simulation, we ran 100 charge–discharge cycles under this accelerated aging setup. To verify that this accelerated aging scheme preserves the relative ranking of charging protocols, we simulated three representative protocols for 1000 cycles without acceleration. The results showed that the relative performance ordering remained consistent across both settings (Appendix E.3). Because all charging protocols must reach the target SOC within a fixed 0.5‐h window, occasional voltage overshoot is unavoidable. Although the degradation model captures some voltage‐driven processes, it does not include all harmful side reactions. We therefore introduce an additional voltage penalty term to account for these unmodeled effects and to promote voltage‐safe charging strategies (Appendix E.4). At the end of each cycle, the state‐of‐health (SOH) is computed as a composite measure combining the dischargeable capacity at the end of cycle i, denoted $Q_{\mathrm{cap}}^{\left(i\right)}$, with the capacity‐equivalent voltage penalty denoted $P^{\left(i\right)}$. The SOH is defined as

(3) $${\mathrm{SOH}}^{\left(i\right)}=\frac{Q_{\mathrm{cap}}^{\left(i\right)}}{Q_{\mathrm{nom}}}-\frac{P^{\left(i\right)}}{Q_{\mathrm{nom}}}$$

with $Q_{\mathrm{nom}}$ representing the nominal cell capacity.

We define the loss function based solely on the SOH after the final cycle. If the final SOH drops below a threshold, a large constant penalty $C_{\mathrm{penalty}}$ is assigned to reject such solutions. Otherwise, the loss decreases smoothly with increasing SOH. Formally, the loss function is defined as:

(4) $$L=\left\{\begin{array}{cc} C_{\mathrm{penalty}}, & \text{if}\;{\mathrm{SOH}}^{\left(N\right)}<0.6\\ -\ln\left(\frac{{\mathrm{SOH}}^{\left(N\right)}-0.6}{1-0.6}\right), & \text{if}\;{\mathrm{SOH}}^{\left(N\right)}\geq 0.6 \end{array}\right.$$

In our work, SOC is defined by Coulomb counting, i.e., by integrating the charging current and normalizing by the nominal capacity $Q_{\text{nom}}$:

(5) $$\mathrm{SOC}\left(t\right)=\mathrm{SOC}\left(0\right)-\frac{1}{Q_{\text{nom}}}\int_{0}^{t}I\left(\tau \right)\;d\tau$$

where I(τ) is the applied current at time τ. This definition is consistent with experimental practice, where SOC cannot be measured directly and is always inferred from the accumulated charge. In each cycle, the cell is first fully discharged (thereby resetting the SOC to zero) and then charged to a fixed target SOC. We set this target to 90% rather than 100% for two considerations. First, as the cell ages and its usable capacity declines, a nominal 100% SOC (defined with respect to the initial rated capacity) no longer corresponds to a true full charge. Therefore, using a slightly reduced target avoids artificially pushing the cell into an overcharged state as capacity fades. Second, terminating the charge below full capacity is a well‐established practice in fast‐charging protocols, as it limits exposure to the high‐voltage regime and thus alleviates voltage‐induced degradation.

To provide a comparative baseline, we implemented a representative multi‐step constant‐current method based on Attia et al. [8]. Specifically, we replaced our LLM‐derived current profile with three consecutive CC segments terminating at 20%, 40%, and 60% SOC, consistent with the protocol class used in that study. This approach maintains the same 0.5 h duration to reach 90% SOC as in our method. We additionally tested higher‐dimensional multi‐step CC parameterizations under the same evaluation budget to examine whether simply increasing the number of fixed CC segments can close the representation gap.

Throughout Case 3, we define the evaluation budget as the number of full protocol evaluations, where one evaluation corresponds to running the complete 100 cycle charge–discharge procedure and computing the final loss. Figure 4 presents the resulting performance comparison. The multi‐step CC baseline and P2P are each allocated 210 evaluations per independent run, with 10 independent runs. For P2O, we report two regimes: (i) a matched‐budget inner‐optimization phase, in which each initial LLM‐generated neural architecture is optimized using the same 210‐evaluation budget; and (ii) an additional outer‐loop phase, where further LLM‐guided architecture evolution is allowed and each new architecture consumes another 210 inner‐loop evaluations. Only regime (i) is a matched‐budget comparison; regime (ii) is included to show how P2O behaves when extra evaluations are available. Figure 5 summarizes the results. Figure 5b depicts selected charge–discharge trajectories of the top‐performing P2O protocol across the 100‐cycle simulation. Figure 5c–e compares the best P2O, P2P, and CCCV protocols, respectively, together with representative best, worst, and intermediate trajectories.

As shown in Figure 5, both P2O and P2P outperform the multi‐step CC baseline in this simulated accelerated‐ageing setting, improving final SOH by 4.24% and 4.17%, respectively. In the matched‐budget phase of Figure 4, P2P achieves the best performance, while P2O already exceeds the CC baseline before any outer‐loop architecture evolution. P2P reaches its final performance within this initial trial budget, whereas P2O attains its best result only after additional architecture‐evolution iterations. With these additional evaluations, P2O ultimately surpasses P2P and attains the best overall performance. The strong performance of P2P in this case is consistent with the structure of its prompt: it explicitly states that exceeding 4.2 V incurs a penalty. This lets the LLM propose candidates that respect the voltage‐safety requirement before numerical evaluation. In contrast, the neural‐network parameterization used in P2O cannot encode such language‐level constraints directly; the behavior must instead be discovered through repeated parameter trials in the inner loop. This result supports a practical distinction between the methods: P2P can be efficient when the key constraints are easy to express in language, whereas P2O is more systematic when numerical tuning of a chosen representation is required.

Inspection of the best‐performing P2P protocol provides a possible physical interpretation of its improved SOH. The policy applies a relatively high charging current early, while SOC and terminal voltage remain low, and progressively reduces the current as the cell enters the high‐SOC/high‐voltage region. This redistributes charging effort toward the lower‐voltage part of the 0–90% SOC window, which can reduce the magnitude and duration of high‐voltage exposure and may reduce reliance on the constrained CC stage near the end of charge. Such behavior is physically plausible because it limits conditions that promote parasitic reactions at high voltage. Within the present model this may reduce SEI‐related and mechanically driven ageing, while the additional voltage penalty also represents unmodeled high‐voltage processes such as electrolyte oxidation and transition‐metal dissolution. Consistently, the best P2P policy in Appendix E.5 depends explicitly on voltage and SOC and suppresses charging current as terminal voltage approaches 4.20 V. We therefore attribute the SOH improvement, at least in part, to shifting charging effort away from the degradation‐sensitive high‐voltage region, while noting that these simulations do not isolate the contribution of individual degradation mechanisms. As in Case 2, the exact policy is not assumed to transfer unchanged across cells, but it can provide a simulation‐informed starting point for related conditions and experimental refinement.

The original multi‐step CC baseline includes only three parameters, which makes it less expressive than our method. To narrow this gap, we increased the number of CC segments to 35, thereby giving the baseline method a comparable number of tunable degrees of freedom. However, this high‐dimensional piecewise‐constant parameterization was difficult to optimize within the same evaluation budget. The resulting SOH trajectories exhibited pronounced sawtooth patterns, as shown in Appendix Figure E2. This behavior highlights a trade‐off in multi‐step CC parameterizations: too few segments restrict the solution space, whereas many segments can be hard to tune and may produce jagged control signals unless additional smoothness constraints or specialized optimizers are introduced.

**FIGURE E1:**
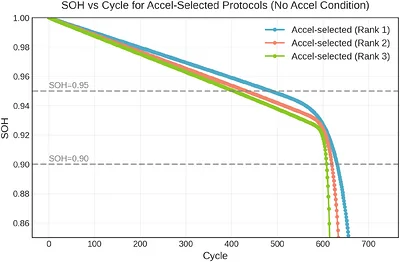
Validation of acceleration factor via long‐term non‐accelerated simulations. To enable observable degradation within limited simulation time, aging was artificially accelerated by increasing the SEI exchange current density by a factor of 5. To verify that this acceleration preserves the relative ranking among charging protocols, three representative protocols selected under accelerated aging were simulated over 1000 cycles under non‐accelerated conditions. The ranking consistency across protocols confirms that the applied acceleration factor preserves protocol differentiation.

## 4 Discussion and Conclusion

Our results answer the motivating question posed in the Introduction: whether LLM‐assisted optimization can remain useful when only a limited number of feedback evaluations is available. The answer is positive, but with an important qualification. The LLM should not generally be viewed as a precise numerical optimizer to replace gradient‐based training, Bayesian optimization, or other dedicated optimization methods. Instead, its main value lies in proposing executable policy representations that broaden the search space beyond fixed, manually specified templates. In the degradation‐aware fast‐charging simulation, this role is sufficient for P2P to achieve strong performance within the matched evaluation budget, improving final SOH by approximately 4.2% relative to a representative multi‐step CC baseline. At the same time, the comparison with P2O shows the benefit of separating representation generation from numerical parameter tuning: when additional evaluations are available, LLM‐generated policy representations can be further refined by SAASBO, providing a more systematic optimization route. Thus, the broader implication is not that LLMs solve battery fast charging directly, but that they can serve as representation generators for limited‐feedback, black‐box scientific optimization problems.

The comparison between P2P and P2O clarifies when each method is useful. P2P is best viewed as a fast population‐based heuristic: the LLM proposes complete executable policies, and the evaluator selects those that perform well. It is therefore attractive when the main design requirements can be expressed clearly in language, as in the voltage‐constrained fast‐charging case. P2O is more conservative: the LLM proposes a compact policy representation, but numerical parameter tuning is delegated to SAASBO. This makes P2O preferable when the prompt does not contain enough physical or structural guidance for direct protocol generation, or when many continuous parameters require systematic tuning. We include both methods because they answer different methodological questions: P2P tests how far direct LLM‐generated protocols can go under a small budget, whereas P2O tests whether LLMs are better used as representation generators coupled to established black‐box optimizers. P2P also tends to produce relatively intricate, problem‐specific expressions that can be difficult to interpret or audit for behaviors such as discontinuities, whereas P2O produces compact neural‐network policies whose internal reasoning is not necessarily interpretable but whose overall functional structure is simpler and more controlled.

Our gradient‐free formulation is complementary to surrogate modeling and differentiable optimal control. Those approaches may be stronger choices when a differentiable simulator, a calibrated reward model, or a large number of safe interactions is available. Although our case studies use simulation for rapid and controlled evaluation, both methods are gradient‐free and are therefore compatible in principle with closed‐loop experimental deployment. While the exact magnitude of gains may vary under measurement noise and model mismatch, the methodological mechanism of representation expansion and language‐specified constraints within a gradient‐free closed loop is, in principle, independent of whether the evaluator is a simulator or a physical cell. When simulations are reasonably accurate, optimization can first be performed in silico and then fine‐tuned on physical batteries. For P2O, simulation helps identify promising neural‐network architectures for the outer loop, after which only the inner‐loop optimization needs to be run experimentally, potentially augmented with multi‐fidelity Bayesian optimization to combine simulation and real data efficiently. For P2P, simulation can be used to update the protocol pool, enabling real experiments to continue improving protocols from a simulation‐informed starting point. These pathways show that LLM‐driven optimization is not only effective in simulation but also well suited for efficient deployment in real‐world scenarios such as battery‐protocol design.

While our approach shares with existing LLM‐based architecture and program search the use of LLM prior knowledge to propose promising candidate structures, battery charging introduces a different optimization regime. Candidate evaluations are relatively slow and expensive, meaning that useful solutions must be identified within a limited number of feedback iterations rather than through thousands of trial‐and‐error evaluations (as deployed in FunSearch). This makes early‐stage optimization performance especially important. It also explains why conventional charging‐protocol optimization often relies on low‐dimensional representations, such as fixed analytical functions or a small number of piecewise‐constant segments: these are easier to optimize under small evaluation budgets, but they restrict the range of behaviors that can be discovered. LLMs can help broaden this range while allowing constraints and design preferences to be expressed directly in natural language.

In future work, we plan to incorporate rich, language‐based feedback rather than relying solely on scalar rewards, for example, description of the issues observed in the charging protocol (e.g., the location of unwanted voltage spikes). Future extensions could also include voltage‐control concepts, such as dynamically targeting internal electrochemical or lithium‐plating overpotentials rather than terminal voltage alone, to further accelerate charging while maintaining safety margins. We hope that the framework presented here offers a promising new direction not only for battery‐protocol optimization, but also provides insights for other domains where experiments are slow, expensive, and consequently challenging to optimize.

## Conflicts of Interest

The authors declares no conflicts of interest.

## Figures and Tables

**FIGURE E2:**
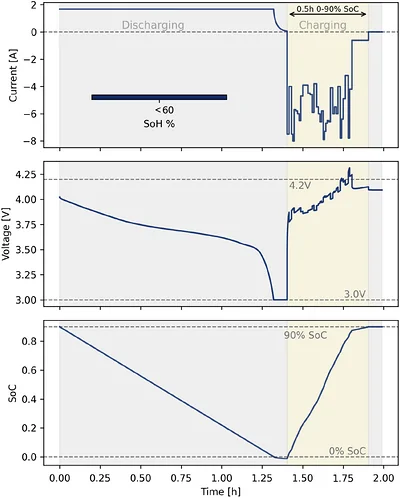
Charging simulation results using the modified multi‐segment CC method with 35 segments. Despite the increased number of parameters, the results exhibit pronounced sawtooth patterns—highlighting the inherent instability of the approach when scaled.

## Data Availability

The data that support the findings of this study are openly available in GitHub at https://github.com/tldr‐group/prompt‐to‐protocol.

## Appendix A SAASBO

Algorithm A1

ALGORITHM A1
saasbooptimize (s,B)
**Table:**

**Require**: Network structure s; BO budget B	
**Ensure**: Best params $\theta^{*}$ and loss $L^{*}$	
1:	Initialise SAAS‐GP surrogate with sparse axis‐aligned prior
2:	Draw an initial Sobol/LHS batch, evaluate ${\left\{\left(\theta_{i},L_{i}\right)\right\}}_{i=1}^{n_{0}}$
3:	**for** k=1 to B **do**
4:	Sample GP hyper‐parameters via MCMC ▹ fits sparse subspace
5:	$\theta_{k}\leftarrow \arg\max_{\theta }$ Acquisition (θ∣SAAS-GP)
6:	$L_{k}\leftarrow \mathrm{TRAINANDEVAL}\left(s,\theta_{k}\right)$
7:	Update SAAS‐GP with $\left(\theta_{k},L_{k}\right)$
8:	$\left(\theta^{*},L^{*}\right)\leftarrow \min_{\;(\theta ,L)}L$ **return** $\theta^{*},\;L^{*}$

## Appendix B Prompts

### B.1 P2P Prompt

Note: Each case below introduces slight variations in the prompt configuration to test the model's ability to adapt the algorithm generation task under different physical and operational conditions.

#### P2P — Case 1

Add to the first line in the constant heating experiment:
to maintain constant heat generation rate. Battery current range: 0–10 A.

Add to the first line in the explicit function experiment:
to match the predefined protocol. Battery current range: 0–10 A.

#### P2P — Case 2

Add to the first line:
to maintain constant heat generation rate as 0.4 W. Battery current range: 0–5 A.

#### P2P — Case 3

Add the following Battery Specifications:
The voltage range should be between 3.0 V and 4.25 V, with an additional penalty applied for operation above 4.20 V. Temperature starts at 308.15 K. Maximum allowed charging current: 8 A. Minimum allowed charging current: 3 A. Charging time should be close to 0.5 h.

#### Base Prompt for P2P Initialization

Generate 10 battery charging control algorithms using PyBaMM with the following constraints:

1. Battery specifications: maximum allowed charging current 10 A; minimum allowed charging current 0 A.
2. The charging current must be negative (e.g., ‐5 means charging at 5 A); in the simulation, negative current indicates charging.
3. The absolute value of the charging current must not exceed 10 A.
4. Do not use any Boolean operators or logic statements such as if, and, or, not, or similar.
5. Use PyBaMM functions and variables only: pybamm.t (time variable), pybamm.sin, pybamm.cos, pybamm.exp, pybamm.tanh, pybamm.sqrt, etc.
6. Do not use pybamm.pi; if you need pi, use 3.14 instead.
7. Try different algorithms; do not use the same or highly similar algorithm multiple times.
8. Each function must be named current_function and must not take any parameters.
9. Each function must return a PyBaMM expression that represents the charging current as a function of time.
10. Make sure every Python code block is enclosed by matching triple backticks and starts with python.

Below is the template for each charging function:

# Charging Function 1
“‘python
def current_function():
t = pybamm.t
# Your algorithm here: build a PyBaMM expression i(t)
return i # i is the charging current expression
“‘

Repeat for Charging Function 2 through 10.

#### Base Prompt for P2P Evolution Tasks

Generate a battery charging control algorithm based on the two given algorithms to maintain a constant heat generation rate using PyBaMM with the following constraints:

1. Battery specifications: maximum allowed charging current 10 A; minimum allowed charging current 0 A.
2. The charging current must be negative (e.g., ‐5 means charging at 5 A); in the simulation, negative current indicates charging.
3. The absolute value of the charging current must not exceed 10 A.
4. Do not use any Boolean operators or logic statements such as “if”, “and”, “or”, “not”, or similar.
5. Use PyBaMM functions and variables only: pybamm.t (time variable), pybamm.sin, pybamm.cos, pybamm.exp, pybamm.tanh, pybamm.sqrt, etc.
6. Do not use pybamm.pi; if you need pi, use 3.14 instead.
7. Try different algorithms; do not use the same or highly similar algorithm multiple times.
8. The function must be named current_function and must not take any parameters.
9. The function must return a PyBaMM expression that represents the charging current as a function of time.
10. Make sure every Python code block is enclosed by matching triple backticks and starts with python.

Follow the format below when generating the algorithm:

# Charging Function
“‘python
def current_function():
t = pybamm.t
# Your algorithm here
return i # where i is the charging current expression
“‘

### B.2 P2O prompt

#### Prompt for P2O Initialization Task (Case1)

Generate 10 reasonable trainable (has parameters to learn) neural network structures using PyTorch with the following constraints:

1. **Framework**:
  Use PyTorch. Import torch, torch.nn, and torch.nn.functional.
2. **Output Scaling**:
  Ensure the final output is scaled to the range [0, 10]. Use a sigmoid activation followed by scaling to achieve this.
3. **Parameters Constraint**:
  Each neural network should have a single input and a single output.
4. **Layer Configuration**:
  - Number of layers should range between 0 and 3.
  - Neurons in each layer should be between 1 and 4.
  - Consider use different activation functions, normalization layers.
  - Do not use dropout layers.
5. **Network Naming**:
  Each generated class should be named NeuralNetwork. When creating instances of these classes, the name NeuralNetwork should always be used.

Below is a template for each network:
**Neural Network 1**

import torch
import torch.nn as nn
import torch.nn.functional as F
class NeuralNetwork(nn.Module):
def __init__(self):
super(NeuralNetwork, self).__init__()
…
def forward(self, input):
…
output = torch.sigmoid(x) * 10 # Scale output to range [0, 10]
return output

Repeat this pattern for 10 different networks.

#### Prompt for P2O Evolution Task (Case1)

Based on the two given neural networks, which represent the shape of a charging protocol, make a small and focused adjustment to generate a new neural network. Follow these specific instructions:

1. **Framework**: Use PyTorch. Import torch, torch.nn, and torch.nn.functional.
2. **Output Scaling**: Ensure the final output is scaled to the range of 0 to 10.
3. **Parameters Constraint**: The new neural network should have a single input and a single output.
4. **Network Structure & Trick**: Try different number of layers and neurons. Incorporate various activation functions, residual connections, and different normalization layers to enhance performance. IMPORTANT: But don't use dropout.
5. **Parameter Number**: The parameters of the generated network should be less than 35.
6. **Structure**: Provide your answer in the following format:

import torchimport torch.nn as nnimport torch.nn.functional as Fclass NeuralNetwork(nn.Module):def __init__(self):super(NeuralNetwork, self).__init__()…def forward(self, input):…output = torch.sigmoid(x) * 10 # Scale output to range [0, 10]return output

#### Prompt for P2O Initialization Task (case3)

You are tasked with generating 10 PyTorch neural networks and their symbolic counterparts in PyBaMM, including RNN, MLP, LSTM, etc. Follow the strict design constraints and code template provided below:

1. **Framework**:
  - You must import:
    import torchimport torch.nn as nnimport torch.nn.functional as Fimport pybammimport numpy as np
2. **Output Scaling**:
  - Ensure the final output is scaled to the range [‐8, ‐3].
  - Use a sigmoid activation followed by scaling to achieve this.
3. **Parameters Constraint**:
  - Each neural network should have three inputs and a single output.
4. **Layer Configuration**:
  - Consider different architectures: MLP, RNN, LSTM, etc.
  - Consider different activation functions.
  - Do not use dropout layers.
  - The total number of parameters of each generated network should be less than 35.
5. **Network Naming**:
  - Each generated PyTorch class should be named NeuralNetwork.
  - The PyBaMM symbolic version should be a standalone function named nn_in_pybamm.

Below is an example and template for each network:
**Neural Network 1**

import torch
import torch.nn as nn
import torch.nn.functional as F
import pybamm
import numpy as np
class NeuralNetwork(nn.Module):
def __init__(self):
super().__init__()
self.fc1 = nn.Linear(3, 3)
self.ln1 = nn.LayerNorm(3, elementwise_affine = False)
self.act = nn.LeakyReLU(0.01)
self.fc2 = nn.Linear(3, 3)
self.fc3 = nn.Linear(3, 1)
def forward(self, x: torch.Tensor) ‐> torch.Tensor:
h = self.fc1(x)
h = self.ln1(h)
h = self.act(h)
h_res = self.fc2(h) + h
h = self.act(h_res)
out = ‐torch.sigmoid(self.fc3(h)) * 5 ‐ 3
return out
def nn_in_pybamm(X, Ws, bs):
W1, W2, W3 = [w.tolist() for w in Ws]
b1, b2, b3 = [b.tolist() for b in bs]
hidden = len(b1)
assert hidden = = 3
# — layer 1: affine —
lin1 = []
for i in range(hidden):
x = pybamm.Scalar(b1[i])
for j in range(3):
x += pybamm.Scalar(W1[i][j]) * X[j]
lin1.append(x)
# — LayerNorm (no affine) —
mean1 = sum(lin1) / hidden
var1 = sum((x ‐ mean1) ** 2 for x in lin1) / hidden
eps = 1e‐5 # small constant to avoid division by zero
inv_std1 = 1 / pybamm.sqrt(var1 + pybamm.Scalar(eps))
ln1 = [(x ‐ mean1) * inv_std1 for x in lin1]
# — activation —
h1 = [pybamm.maximum(x, 0.01 * x) for x in ln1]
# — layer 2: affine + residual + activation —
lin2 = []
for i in range(hidden):
x = pybamm.Scalar(b2[i])
for j in range(hidden):
x += pybamm.Scalar(W2[i][j]) * h1[j]
lin2.append(x)
h2 = [
pybamm.maximum(lin2[i] + h1[i], 0.01 * (lin2[i] + h1[i]))
for i in range(hidden)
]
# — output layer + sigmoid + scaling —
lin3 = pybamm.Scalar(b3[0])
for j in range(hidden):
lin3 += pybamm.Scalar(W3[0][j]) * h2[j]
y = 1 / (1 + pybamm.exp(‐lin3))
I_scale = 5 # Scale output to current range
return ‐pybamm.Scalar(I_scale) * y ‐ 3

**Neural Network 2**

import torch
import torch.nn as nn
import torch.nn.functional as F
import pybamm
import numpy as np
class NeuralNetwork(nn.Module):
def __init__(self):
super(NeuralNetwork, self).__init__()
…
def forward(self, input):
…
output = ‐torch.sigmoid(x) * 5 ‐ 3
# Scale output to range [‐8, ‐3]
return output
def nn_in_pybamm(X, Ws, bs):
…
return ‐pybamm.Scalar(I_scale) * y ‐ 3

Repeat this pattern for 10 different networks.

#### Prompt for P2O Evolution Task (Case3)

You are tasked with generating a PyTorch neural network and its symbolic counterpart in PyBaMM, based on two given example networks. Follow the strict design constraints and code template provided below:

1. **Framework**:
  - You must import:
    import torchimport torch.nn as nnimport torch.nn.functional as Fimport pybammimport numpy as np
2. **Output Scaling**:
  - Ensure the final output is scaled to the range [‐8, ‐3].
  - Use a sigmoid activation followed by scaling to achieve this.
3. **Parameters Constraint**:
  - Each neural network should accept three input features per time step and produce a single scalar output.
4. **Layer Configuration**:
  - Consider different architectures (e.g., MLP, RNN, LSTM, etc.).
  - Do not use dropout layers.
  - Vary activation functions (e.g., ReLU, LeakyReLU, ELU, Tanh, etc.).
  - You may include residual (skip) connections.
  - The total number of parameters of each generated network should be less than 35.
5. **Naming**:
  - The PyTorch model class must be named NeuralNetwork.
  - The PyBaMM symbolic function must be named nn_in_pybamm.

Below is the template to follow for each generated network:

import torch
import torch.nn as nn
import torch.nn.functional as F
import pybamm
import numpy as np
class NeuralNetwork(nn.Module):
def __init__(self):
super().__init__()
# define layers, layer norms, activations here
def forward(self, x: torch.Tensor) ‐> torch.Tensor:
# forward through layers, activations, residuals here
out = torch.sigmoid(self.final_layer(x)) * 5 ‐ 3
return out
def nn_in_pybamm(X, Ws, bs):
# Unpack weights and biases
# Build symbolic computation
return ‐pybamm.Scalar(5) * y ‐ 3

## Appendix C Case Study 1

### C.1 ECM Model

The heat generation Q and temperature update T at each time step are calculated using the following ECM‐based equations:

(C1)
$$\begin{array}{cc} R_{0} & =R_{0,\text{ref}}\left(1+\alpha (T-T_{\text{ref}})\right) \end{array}$$

(C2)
$$\begin{array}{cc} R_{p} & =R_{p,\text{ref}}\left(1+\alpha (T-T_{\text{ref}})\right) \end{array}$$

(C3)
$$\begin{array}{cc} Q & =I^{2}\left(R_{0}+R_{p}\right) \end{array}$$

(C4)
$$\begin{array}{cc} T_{\text{next}} & =T+\Delta t\cdot \frac{I^{2}\left(R_{0}+R_{p}\right)-hA\left(T-T_{\text{ambient}}\right)}{mc} \end{array}$$

**Where**:

- I: current input [A]
- T: current temperature [K]
- $R_{0}$, $R_{p}$: ohmic and polarization resistances [Ω]
- $R_{0,\text{ref}}$, $R_{p,\text{ref}}$: reference resistances at $T_{\text{ref}}$ [Ω]
- α: temperature coefficient [1/K]
- Q: heat generated [W]
- h: heat transfer coefficient [W/$m^{2}\cdot K$]
- A: surface area for heat exchange [$m^{2}$]
- $T_{\text{ambient}}$: ambient temperature [K]
- m: thermal mass [kg]
- c: specific heat capacity [J/kg·K]
- Δt: time step duration [s]

### C.2 Outer Loop Evaluation Setup

To ensure fair and efficient comparison among the three architecture search methods, we chose distinct but related search ranges tailored to each algorithm's strengths and computational characteristics:

- **Bayesian Optimization (BO)** Operates on a fixed, globally modeled search space with higher per‐sample overhead.
  – Hidden layers: {0,1,2,3,4}
  – Neurons per layer: {1,2,3,4,5}
  – Activation functions: {ReLU, Sigmoid, Tanh, LeakyReLU, ELU, Softplus, Softsign, SiLU, GELU}
  – Normalization: {none, BatchNorm1d, LayerNorm}
  – Optimization backend: Optuna (default TPESampler, no pruning).
- **Evolutionary Algorithm (EA)** Explores architectures progressively via mutation and crossover, benefiting from a narrower initialization that can expand through evolution.
  – Initial hidden layers: {0,1,2,3}
  – Initial neurons per layer: {1,2,3,4}
  – Activation functions (initial and mutation): {ReLU, Sigmoid, Tanh, LeakyReLU, ELU, Softplus, Softsign, SiLU, GELU}
  – Normalization (initial and mutation): {none, batchnorm, layernorm}
  – Mutation probability: $p_{\mathrm{mut}}=0.1$
  – Mutation operators:
    * Width perturbation: ±1 neuron, clipped to [1,10]
    * Activation replacement among the nine choices
    * Normalization add/remove/change
  – Crossover: single‐point on layer sequence when both parents have ≥2 layers; otherwise local width perturbation
  – Selection: tournament size = 2
- **Random Sampling** Serves as a non‐adaptive baseline, drawing architectures independently each generation from the same initial space as EA for direct comparison of adaptive benefits.
  – Hidden layers: {0,1,2,3}
  – Neurons per layer: {1,2,3,4}
  – Activation functions: {ReLU, Sigmoid, Tanh, LeakyReLU, ELU, Softplus, Softsign, SiLU, GELU}
  – Normalization: 20% chance per layer to apply either batchnorm or layernorm
  – Generation size: 3 new models per generation, over 150 generations
- **Unified Parameter Cap** To prevent over‐parameterization and ensure comparability, all methods enforced a hard limit of 35 trainable parameters. Any architecture exceeding this threshold was assigned a fixed loss of $1\times 10^{6}$ during evaluation.

### C.3 P2P Optimal Algorithms

**Python implementation**

def current_function():
t = pybamm.t
# RMS‐coherence regulator
# Smooth progress variables
q = t / (t + 680)
s = 0.5 * (1 + pybamm.tanh((t ‐ 320) / 560))
w = pybamm.sqrt(1 + t / 820)
# Phase carriers
phi1 = t / (540 + 130 * q) +
0.19 * pybamm.sin(2 * 3.14 * t / (930 + 190 * w))
phi2 = t / (780 + 210 * w) ‐
0.16 * pybamm.cos(2 * 3.14 * t / (670 + 210 * q))
phi3 = t / (1180 + 280 * s) +
0.12 * pybamm.sin(2 * 3.14 * t / (960 + 260 * s))
u = pybamm.sin(2 * 3.14 * phi1)
v = pybamm.cos(2 * 3.14 * phi2)
z = pybamm.sin(2 * 3.14 * phi3)
# RMS amplitude and cross‐coherence
A = pybamm.sqrt((u * u + v * v + z * z) / 3)
C = (u * v + v * z + z * u) / 3
# Composite coherence metric
H = 0.55 * A + 0.45 * pybamm.exp(‐0.7 * C * C)
# Ripple budget
r = 0.017 + 0.011 * q * (0.5 + 0.5 * s)
# Near‐constant magnitude with smooth, bounded ripple
M = 0.90
g = M * (1 ‐ r) + M * r / (1 + 0.85 * H + 0.15 * A * A)
# Negative charging current, |i| <= 10 A
i = ‐10 * g
return i

### C.4 Case1 P2O Optimal Neural Network

Figure C1 visualizes the functional priors (search spaces) of the three architectures evolved by the LLM in the constant‐heating search. The Gated Mixed‐Op Network (Figure C1a) is structurally more complex, incorporating multiple gating paths and mixed operations, which results in a visibly richer and more intricate search space. The Linear‐BN‐ReLU Baseline (Figure C1b) follows a more conventional feed‐forward design and therefore exhibits a simpler, although still relatively large, search space. In contrast, the Residual SELU‐Leaky Block (Figure C1c) exhibits a much more constrained and smooth functional prior. Importantly, the optimal protocol in the constant‐heating case follows a similarly simple pattern, gradually decreasing and then stabilizing, as shown in Figure 2f. The functional forms favored by this block align well with the structure of the target solution, which is consistent with the strong optimization performance observed in this setting.

**Neural Network Structure 1**

class NeuralNetwork(nn.Module):
def __init__(self):
super(NeuralNetwork, self).__init__()
# Early stack (from both seeds)
self.fc1 = nn.Linear(1, 4)
self.bn1 = nn.BatchNorm1d(4) # Seed1‐style affine BN
self.ln1 = nn.LayerNorm(4, elementwise_affine=False)
# Blending scalars: decoupled norms (Seed2) + act + tail
self.alpha_act = nn.Parameter(torch.tensor(0.5))
self.alpha_norm1 = nn.Parameter(torch.tensor(0.5))
self.alpha_norm2 = nn.Parameter(torch.tensor(0.5))
self.alpha_tail = nn.Parameter(torch.tensor(0.5))
# Scalar projection (Seed2‐style BN2 affine=False)
self.merge = nn.Linear(4, 1)
self.bn2 = nn.BatchNorm1d(1, affine=False)
self.ln2 = nn.LayerNorm(1, elementwise_affine=False)
# Gating and direct path (common)
self.gate = nn.Linear(1, 1)
self.direct = nn.Linear(4, 1)
# Nonlinearity and final head
self.leaky = nn.LeakyReLU(0.2)
self.out = nn.Linear(1, 1)
def forward(self, input):
w_act = torch.sigmoid(self.alpha_act)
w_n1 = torch.sigmoid(self.alpha_norm1)
w_n2 = torch.sigmoid(self.alpha_norm2)
w_tail = torch.sigmoid(self.alpha_tail)
# Early projection + blended normalization + blended activation
x = self.fc1(input)
x = (1.0 ‐ w_n1) * self.bn1(x) + w_n1 * self.ln1(x)
x = (1.0 ‐ w_act) * F.relu(x) + w_act * F.silu(x)
# Scalar path with separate BN/LN blend
s = self.merge(x)
s = (1.0 ‐ w_n2) * self.bn2(s) + w_n2 * self.ln2(s)
# Input‐conditioned gate and direct injection
z = self.direct(x)
g = torch.sigmoid(self.gate(input))
s_tilde = s + g * z
# Combine Seed1's dual‐tail scheme with Seed2's tail mixer
w_mix_seed1 = 0.5 * (g + w_act)
y1 = (1.0 ‐ w_mix_seed1) * torch.tanh(s_tilde)
+ w_mix_seed1 * self.leaky(s_tilde)
y2 = (1.0 ‐ g) * torch.tanh(s_tilde) + g * self.leaky(s_tilde)
# Crossfade between tails using both norm2 and tail mixers
w_cf = 0.5 * (w_tail + w_n2)
y = (1.0 ‐ w_cf) * y1 + w_cf * y2
# Final mapping and scaling to [0, 10]
x = self.out(y)
output = torch.sigmoid(x) * 10
return output

**Neural Network Structure 2**

class NeuralNetwork(nn.Module):
def __init__(self):
super(NeuralNetwork, self).__init__()
self.fc1 = nn.Linear(1, 3, bias=False)
self.bn1 = nn.BatchNorm1d(3)
self.fc2 = nn.Linear(3, 1, bias=False)
def forward(self, input):
x = self.fc1(input)
x = self.bn1(x) # Batch normalization
x = torch.relu(x) # Using ReLU activation function
x = self.fc2(x)
output = torch.sigmoid(x) * 10 # Scale output to range [0, 10]
return output

**Neural Network Structure 3**

class NeuralNetwork(nn.Module):
def __init__(self):
super(NeuralNetwork, self).__init__()
self.fc1 = nn.Linear(1, 3)
self.fc2 = nn.Linear(3, 3)
self.bn2 = nn.BatchNorm1d(3)
self.out = nn.Linear(3, 1)
self.res_scale = nn.Parameter(torch.tensor(1.0))
def forward(self, input):
h1 = F.selu(self.fc1(input))
h2 = self.fc2(h1)
h2 = self.bn2(h2)
h2 = F.leaky_relu(h2, negative_slope=0.1)
x = h2 + self.res_scale * h1
x = self.out(x)
output = torch.sigmoid(x) * 10
return output

## Appendix D Case study 2

### D.1 P2O Optimal Neural Network (Case 2)

**Neural Network Structure**

import torch
import torch.nn as nn
import torch.nn.functional as F
class NeuralNetwork(nn.Module):
def __init__(self):
super(NeuralNetwork, self).__init__()
self.fc1 = nn.Linear(1, 3, bias=False)
self.bn1 = nn.BatchNorm1d(3)
self.fc2 = nn.Linear(3, 1, bias=False)
def forward(self, input):
x = self.fc1(input)
x = self.bn1(x) # Batch normalization
x = torch.relu(x) # Using ReLU activation function
x = self.fc2(x)
output = torch.sigmoid(x) * 10 # Scale output to range [0, 10]
return output

### D.2 P2P Optimal Algorithm (Case 2)

**Python implementation**

def current_functionx():
t = pybamm.t
Q = 0.4 # target heat (watts)
R0 = 0.02 # base ohmic resistance (ohms)
# Smooth bases
s1 = pybamm.sin(2 * 3.14 * t / 2600 + 0.3)
c1 = pybamm.cos(2 * 3.14 * t / 3900 ‐ 0.6)
s2 = pybamm.sin(2 * 3.14 * t / 5200 + 1.0)
c2 = pybamm.cos(2 * 3.14 * t / 6700 + 0.8)
s3 = pybamm.sin(2 * 3.14 * t / 8100 ‐ 1.1)
c3 = pybamm.cos(2 * 3.14 * t / 9500 + 0.2)
# Nonlinear modulators
u1 = pybamm.tanh(0.7 * s1 + 0.4 * c2)
u2 = pybamm.tanh(0.6 * s2 ‐ 0.5 * c1)
u3 = pybamm.tanh(0.5 * s3 + 0.6 * c3)
u4 = pybamm.tanh(0.8 * s1 ‐ 0.3 * c3)
# Positive, time‐varying resistances (diverse constructions)
R1 = R0 * pybamm.exp(0.22 * s1 + 0.10 * c1 + 0.06 * s2 * c2)
R2 = R0 * (1.05 + 0.25 * s2 + 0.15 * c2 + 0.10 * u1) ** 2
R3 = R0 * pybamm.exp(0.18 * c3 ‐ 0.08 * s1 + 0.05 * u2)
R4 = R0 * (0.95 + 0.20 * c1 ‐ 0.10 * s3 + 0.20 * u3) ** 2
R5 = R0 * pybamm.exp(0.14 * u4 + 0.07 * s2 ‐ 0.04 * c2)
R6 = R0 * (1.00 + 0.18 * s1 * c1 + 0.12 * s2 * s3) ** 2
# Smooth softmax weights over six channels
gamma = 1.0 + 0.3 * pybamm.sin(2 * 3.14 * t / 10400)
v1 = pybamm.exp(gamma * s1)
v2 = pybamm.exp(gamma * c1)
v3 = pybamm.exp(gamma * s2)
v4 = pybamm.exp(gamma * c2)
v5 = pybamm.exp(gamma * s3)
v6 = pybamm.exp(gamma * c3)
sumv = v1 + v2 + v3 + v4 + v5 + v6
w1 = v1 / sumv
w2 = v2 / sumv
w3 = v3 / sumv
w4 = v4 / sumv
w5 = v5 / sumv
w6 = v6 / sumv
# Tri‐mean over arithmetic, geometric, and harmonic means (A‐G‐H)
R_arith = w1 * R1 + w2 * R2 + w3 * R3 + w4 * R4 + w5 * R5 + w6 * R6
R_harm =
1 / (w1 / R1 + w2 / R2 + w3 / R3 + w4 / R4 + w5 / R5 + w6 / R6)
R_geom = pybamm.exp(
w1 * pybamm.log(R1)
+ w2 * pybamm.log(R2)
+ w3 * pybamm.log(R3)
+ w4 * pybamm.log(R4)
+ w5 * pybamm.log(R5)
+ w6 * pybamm.log(R6)
)
delta = 0.7
x1 = pybamm.exp(delta * pybamm.sin(2 * 3.14 * t / 7200 + 0.4))
x2 = pybamm.exp(delta * pybamm.cos(2 * 3.14 * t / 7800 ‐ 0.9))
x3 = pybamm.exp(delta * pybamm.sin(2 * 3.14 * t / 8400 + 1.1))
sumx = x1 + x2 + x3
a = x1 / sumx
b = x2 / sumx
c = x3 / sumx
R_AHG = (R_arith ** a) * (R_geom ** b) * (R_harm ** c)
# Conductance‐side power‐harmonic mean, mapped back to resistance
G1 = 1 / R1
G2 = 1 / R2
G3 = 1 / R3
G4 = 1 / R4
G5 = 1 / R5
G6 = 1 / R6
r = 0.6 + 0.4 * pybamm.sin(2 * 3.14 * t / 8800 ‐ 0.2)
denom = (
w1 / (G1 ** r)
+ w2 / (G2 ** r)
+ w3 / (G3 ** r)
+ w4 / (G4 ** r)
+ w5 / (G5 ** r)
+ w6 / (G6 ** r)
)
G_ph = (1 / denom) ** (1 / r)
R_condpath = 1 / G_ph
# Harmonic blending between A‐G‐H path and conductance path
alpha = 0.5 + 0.5 * pybamm.sin(2 * 3.14 * t / 7600 + 0.3)
R_tilde = 1 / (alpha / R_AHG + (1 ‐ alpha) / R_condpath)
# Smooth envelope to diversify dynamics
env = pybamm.exp(0.12 *
pybamm.tanh(0.5 * s1 ‐ 0.4 * c3 + 0.3 * s2 * c1))
R_t = R_tilde * env
# Current targeting constant heat:
# i^2 * R_t \approx Q, saturated to [‐5, 0] A
i_mag = pybamm.sqrt(Q / R_t)
i = ‐5 * pybamm.tanh(i_mag / 5)
return i

## Appendix E Case Study 3

### E.1 Degradation Submodels

The following degradation submodels were enabled in PyBaMM for the DFN simulations:

- **Particle**: Fickian diffusion
- **Particle mechanics**: swelling and cracking
- **Loss of active material**: stress‐driven
- **SEI**: reaction‐limited
- **SEI porosity change**: true
- **SEI on cracks**: true
- **Thermal model**: lumped
- **Discharge energy calculation**: enabled

### E.2 Accelerated Aging Parameter Modifications

To accelerate degradation, several parameters were modified relative to the default Ai2020 values. These are summarized in Table E1.

**TABLE E1:** Accelerated aging parameters (relative to Ai2020 baseline).

Parameter	Modified value
SEI reaction exchange current density [A $m^{-2}$]	×5
Negative electrode LAM constant proportional term [$s^{-1}$]	$1.0\times 10^{-12}$
Positive electrode LAM constant proportional term [$s^{-1}$]	$2.78\times 10^{-13}\times 10$
Positive electrode cracking rate	$3.9\times 10^{-20}\times 10$
Negative electrode cracking rate	$3.9\times 10^{-20}\times 10$
Total heat transfer coefficient [W $m^{-2}$ $K^{-1}$]	5.0
Ambient temperature [K]	308.15

### E.3 Validation of Accelerated Aging

To verify that accelerated aging preserves the relative ranking of charging protocols, we repeated simulations for three representative protocols under non‐accelerated conditions for 1000 cycles. Results confirmed that protocol rankings were consistent between accelerated and non‐accelerated cases (see Figure E1).

### E.4 Penalty on High Voltage

(E1)
$$P^{\left(i\right)}\;=\;\alpha \int_{0}^{t_{f}^{\left(i\right)}}{\left[\max\;\left(V\left(t\right)-V_{\mathrm{thr}},\;0\right)\right]}^{n}\;dt,$$

where $V_{\mathrm{thr}}=4.20\;V$, n=3 controls the steepness of the penalty, and α=0.3 is a calibrated scaling factor. The penalty term strongly discourages protocols that sustain voltages above 4.20,V, thereby biasing the optimization toward voltage‐safe strategies. This penalty does not correspond to a directly measured capacity fade; instead, it serves as a surrogate degradation metric introduced to guide the optimization. From a physical perspective, sustained exposure to high voltages accelerates parasitic side reactions such as electrolyte oxidation and transition–metal dissolution, which are known to contribute to long‐term capacity loss. Therefore, penalizing the duration and magnitude of voltage overshoot provides a practical proxy for discouraging degradation‐prone charging behaviors. At the end of each cycle, the state‐of‐health (SOH) is updated by combining the measured capacity $Q_{\mathrm{cap}}^{\left(i\right)}$ with this penalty term:

(E2)
$${\mathrm{SOH}}^{\left(i\right)}\;=\;\frac{Q_{\mathrm{cap}}^{\left(i\right)}-P^{\left(i\right)}}{Q_{\text{nom}}},$$

### E.5 P2P Optimal Algorithm (Case 3)

**Python implementation**

def adaptive_current(vars_dict):
V = vars_dict[“Voltage [V]”]
T = vars_dict[“Volume‐averaged cell temperature [K]”]
SOC = vars_dict[“SoC”]
# Smoothstep shaping in V and SoC (cubic 3x^2 ‐ 2x^3)
x_v = pybamm.minimum(1, pybamm.maximum(0, 0.5 + (4.08 ‐ V) / 0.35))
s_v = x_v**2 * (3 ‐ 2 * x_v)
x_s = pybamm.minimum(1, pybamm.maximum(0, 0.5 + (0.60 ‐ SOC) / 0.50))
s_s = x_s**2 * (3 ‐ 2 * x_s)
base = 0.55 * s_v + 0.45 * s_s + 0.50
* s_v * s_s ‐ 0.12 * s_v * (1 ‐ s_s)
base_clamped = pybamm.minimum(1, pybamm.maximum(0, base))
shape = 1 ‐ pybamm.exp(‐2.6 * base_clamped)
# Temperature modulation: Gaussian around 308.15 K + mild sinusoid
zT = (T ‐ 308.15) / 17
t_fac = 0.92 + 0.20 * pybamm.exp(‐(zT ** 2))
+ 0.06 * pybamm.sin((T ‐ 308.15) / 22)
# Voltage penalties above 4.20 V and near 4.25 V (softplus hinges)
v_soft = pybamm.log(1 + pybamm.exp(70 * (V ‐ 4.20))) / 70
v_pen1 = pybamm.exp(‐90 * v_soft) / (1 + 25 * v_soft) + 0.04
v_cap = pybamm.log(1 + pybamm.exp(140 * (V ‐ 4.25))) / 140
v_pen = v_pen1 * pybamm.exp(‐160 * v_cap)
# Desired magnitude and clamp
desired = (3.0 + 6.6 * shape) * t_fac * v_pen
mag = pybamm.minimum(8, pybamm.maximum(3, desired))
return ‐mag

### E.6 P2O Optimal Neural Network (Case 3)

**Python implementation**

import torch
import torch.nn as nn
import torch.nn.functional as F
import pybamm
import numpy as np
class NeuralNetwork(nn.Module):
def __init__(self):
super().__init__()
# Tiny MLP with a residual skip from input to output
self.fc1 = nn.Linear(3, 2, bias=True) # 8 params
self.ln1 = nn.LayerNorm(2, elementwise_affine=False) # 0 params
self.fc_out = nn.Linear(2, 1, bias=True) # 3 params
self.skip = nn.Linear(3, 1, bias=False) # 3 params
# Total trainable params: 14 (< 35)
def forward(self, x: torch.Tensor) ‐> torch.Tensor:
h = self.fc1(x)
h = self.ln1(h)
h = torch.tanh(h)
y_main = self.fc_out(h)
y = y_main + self.skip(x) # residual connection
out = ‐torch.sigmoid(y) * 5 ‐ 3 # scale to [‐8, ‐3]
return out
def nn_in_pybamm(X, Ws, bs):
“””
Build the symbolic counterpart in PyBaMM.
Arguments:
‐ X: list‐like of 3 PyBaMM symbols [x0, x1, x2]
‐ Ws: [W1(2x3), W2(1x2), Wskip(1x3)]
‐ bs: [b1(2), b2(1)]
“’'”
W1, W2, Wskip = [w.tolist() for w in Ws]
b1, b2 = [b.tolist() for b in bs]
# z1 = W1 * X + b1 ‐> shape (2,)
z1 = []
for i in range(2):
zi = pybamm.Scalar(b1[i])
for j in range(3):
zi += pybamm.Scalar(W1[i][j]) * X[j]
z1.append(zi)
# LayerNorm over 2 features (no affine):
# (z ‐ mean) / sqrt(var + eps)
mean1 = (z1[0] + z1[1]) / 2
var1 = ((z1[0] ‐ mean1) ** 2 + (z1[1] ‐ mean1) ** 2) / 2
inv_std1 = 1 / pybamm.sqrt(var1 + pybamm.Scalar(1e‐5))
h1 = [(z1[i] ‐ mean1) * inv_std1 for i in range(2)]
# Activation: tanh
a1 = [pybamm.tanh(h1[i]) for i in range(2)]
# Main head: y_main = W2 * a1 + b2
y_main = pybamm.Scalar(b2[0])
for i in range(2):
y_main += pybamm.Scalar(W2[0][i]) * a1[i]
# Residual skip: y_skip = Wskip * X (no bias)
y_skip = pybamm.Scalar(0)
for j in range(3):
y_skip += pybamm.Scalar(Wskip[0][j]) * X[j]
# Combine and scale with sigmoid to [‐8, ‐3]
y_lin = y_main + y_skip
y_sig = 1 / (1 + pybamm.exp(‐y_lin))
return ‐pybamm.Scalar(5) * y_sig ‐ 3
